# Most Trial Eligibility Criteria and Patient Baseline Characteristics Do Not Modify Treatment Effect in Trials Using Targeted Therapies for Rheumatoid Arthritis: A Meta-Epidemiological Study

**DOI:** 10.1371/journal.pone.0136982

**Published:** 2015-09-11

**Authors:** Anton Wulf Christensen, Simon Tarp, Daniel E. Furst, Anna Døssing, Kirstine Amris, Henning Bliddal, Peter C. Taylor, Robin Christensen

**Affiliations:** 1 The Parker Institute, Department of Rheumatology, Copenhagen University Hospitals, Bispebjerg and Frederiksberg, Frederiksberg, Denmark; 2 David Geffen School of Medicine, University of California in Los Angeles, Los Angeles, California, United States of America; 3 Kennedy Institute of Rheumatology: Nuffield Department of Orthopaedics, Rheumatology and Musculoskeletal Sciences, University of Oxford, Botnar Research Centre, United Kingdom; University of Texas Health Science Center at Houston, UNITED STATES

## Abstract

**Objective:**

To determine if variations in trial eligibility criteria and patient baseline characteristics could be considered effect modifiers of the treatment response when testing targeted therapies (biological agents and targeted synthetic disease modifying antirheumatic drugs (DMARDs)) for rheumatoid arthritis (RA).

**Methods:**

We conducted a meta-epidemiological study of all trials evaluating a targeted therapy approved by regulatory authorities for treating RA. The database search was completed on December 11^th^ 2013. Eligible trials reported ACR20 data at months 3–6 and used an add-on design. Odds ratios (ORs) were calculated from the response rates and compared among the trial eligibility criteria/patient baseline characteristics of interest. Comparisons are presented as the Ratio of Odds Ratios (ROR).

**Results:**

Sixty-two trials (19,923 RA patients) were included in the primary analyses using ACR20 response. Overall, targeted therapies constituted an effective treatment (OR 3.96 95% confidence interval (CI) 3.41 to 4.60). The majority of the trial eligibility criteria and patient baseline characteristics did not modify treatment effect. The added benefit of targeted therapies was lower in trials including "DMARD-naïve" patients compared with trials including "DMARD inadequate responders" (ROR = 0.45, 95%CI 0.31 to 0.66) and trials including "targeted therapy inadequate responders" (0.50, 95%CI 0.29 to 0.87), test for interaction: p = 0.0002. Longer mean disease duration was associated with a higher likelihood of responding to treatment (β = 1.05, 95%CI 1.00 to 1.11 OR’s per year; p = 0.03). Analyses conducted using DAS28-remission as the outcome supported the above-mentioned findings.

**Conclusion:**

Our results suggest that a highly selective inclusion is not associated with greater treatment effect, as might otherwise be expected. The added benefit of a targeted therapy was lower in trials including patients who were DMARD-naïve and trials including patients with shorter disease durations.

## Introduction

Newer drugs, consisting of biological disease modifying antirheumatic drugs (bDMARDs) [1] as well as targeted synthetic DMARDs (tsDMARDs) such as agents targeting janus kinases [2] (JAK-inhibitors), are considered effective for treating rheumatoid arthritis (RA) but are also expensive. These drugs, which we will refer to as targeted therapies, are generally recommended in patients with inadequate response to combination treatment with conventional synthetic DMARDs (csDMARDs) [3].

Randomized controlled trials (RCTs) that have tested targeted therapies vary in several of their trial eligibility criteria and patient baseline characteristics [4], but little is known about whether these differences influence the overall treatment effect. Several patient baseline characteristics have previously been explored [5–9], but only the trial participants’ mean disease duration was statistically significant and reproducibly associated with improved outcomes [5;7].

It is unknown if the difference in benefit from therapy depends on whether the outcome of choice is a measure of change such as the ACR20 response criteria (a 20% reduction in the number of swollen and tender joints and 3/5 other core items) [10] or the number of patients reaching a certain threshold representing low disease activity (e.g., DAS28-remission) [11]. Knowledge about whether various trial eligibility criteria or patient baseline characteristics modify treatment effect may lead to a better understanding of the importance of trial design, which is important for clinicians, policy makers, and the pharmaceutical industry alike. Furthermore, knowing whether certain variables and contextual factors act as effect modifiers can also be important for prognostic and health economic reasons and could thus also influence clinical guidelines with regards to optimizing health or economic benefit.

The objective of this study was to examine if variations in trial eligibility criteria and patient baseline characteristics can influence the added benefit of targeted therapies compared to the control treatment in RA trials (i.e., be an effect modifier).

## Method

### Protocol and registration

The protocol describing the study eligibility criteria, data extraction, and analysis was specified in advance and registered at the international prospective register of systematic reviews–PROSPERO (Registration no. CRD42014010322). The study findings are reported according to the Preferred Reporting Items for Systematic reviews and Meta-analyses [12].

### Eligibility criteria

Eligible trials were RCTs of RA [13;14]. The interventions of interest were targeted therapies with standard routes of administration and dosages that were approved by the European Medicines Agency (EMA) and the US Food and Drug Administration (FDA) for treating RA [15;16]. Studies testing anakinra were not included because it is generally accepted to be less effective in RA than other biologic agents [3;17]. Included trials had to be designed with the add-on of a targeted therapy (e.g., bDMARD and MTX vs. MTX alone); studies without an add-on, non-inferiority trials, and biologics head-to-head designs were excluded, as a contrast between these arms would imply something different from our objective. Eligible studies had to report ACR20 response data [10] at month 3–6 or these data had to be available from other sources (e.g., clinicaltrials.gov or later publications). Open-label studies were excluded because they have an inherent risk of performance bias [18].

### Information sources and search strategy

We searched PubMed, EMBASE, The Cochrane Central Register of Controlled Trials (CENTRAL), and LILACS and used a combination of keywords and text words. Our search strategy has previously been published [19]. The search was completed December 11^th^ 2013. The World Health Organization (WHO) Clinical trials Portal (ICTRP), clinicaltrials.gov, FDA, and EMA were searched to identify unpublished data.

### Study selection

Fulfillment of the study eligibility criteria was assessed by two independent reviewers. Titles and abstracts were first screened and duplicates were removed. If one reviewer thought a study could potentially be eligible for inclusion the paper was retrieved and read in full text. Disagreements were resolved by discussion until consensus was reached.

### Data collection process

Data were extracted from trials, using a pre-specified form, by one investigator and subsequently checked by a second investigator. Disagreements were resolved by consensus. Data were extracted from only one active arm (the one using the FDA/EMA approved dosage) and one comparator arm per trial to avoid splitting the comparator group. We extracted outcome data according to the study’s primary outcome time point; if the primary outcome came later than six months into the study, we extracted available data closest to month three.

### Data items and risk of bias in individual studies

Data on the following trial eligibility criteria were extracted: first author, drug of interest, treatment given in the active and comparator arm, minimum required 66 swollen joint count (SJC), minimum required 68 tender joint count (TJC), minimum required C-reactive protein (CRP), maximum allowed disease duration and rheumatoid factor (RF), and/or anti-cyclic citrullinated peptide (anti-CCP) antibody status. Information regarding the patients’ background DMARD medication was also extracted. For "MTX," "csDMARDs (non-MTX csDMARDs),” and "Targeted therapies," we extracted information about how the medication was handled at randomization and grouped the trials into one of the following categories: Continued (i.e., patients were allowed to continue their background medication), Discontinued (i.e., patients were not allowed to continue their background medication), Not using (i.e., the study only included patients who were currently not using the drug(s) of interest), Naïve (i.e., patients had never used the drug(s) of interest, and Not reported (i.e., no information was reported on this matter).

In order to stratify trials according to the DMARD history of their included patients, we asked ourselves the question: Had the participants, prior to inclusion, exhausted the treatment potential of at least one DMARD (or targeted therapy). Trials were then grouped into one of the following categories: DMARD-naïve (patients were either csDMARD naïve or had not exhausted the treatment potential of at least one csDMARD); DMARD-IR (DMARD inadequate responders), where patients had exhausted at least one csDMARD option and had inadequate response); and TT-IR (patients had experienced an inadequate response to at least one previous targeted therapy).

Data on the following patient baseline characteristics were extracted: female%, age, disease duration, CRP, Disease activity score based on 28 joint counts (DAS28), RF%, anti-CCP positive%, SJC, TJC, health assessment questionnaire—disability index (HAQ), MD global assessment of disease activity, patient global assessment of disease activity and patient-reported pain on visual analogue scales of 0–100 mm (VAS). If both median and mean values were given, the former was prioritized. Primary outcome was ACR20 response, as this was most frequently reported. As a secondary outcome, DAS28-remission (DAS28<2.6) was extracted.

The risk of bias (RoB) within each trial was assessed using the RoB tool as recommended by The Cochrane Collaboration [18;20]. Each domain was rated as "low," "high," or "unclear" RoB. Domains were rated as unclear if they failed to meet the criteria for high or low RoB. Two reviewers (ST, AD) independently evaluated RoB. Disagreements were resolved by discussion.

### Synthesis of results

For each trial we estimated the odds ratio (OR) and the corresponding standard error (i.e., log_*e*_OR and SE(log_*e*_OR), respectively) based on the intra-trial contrast between active intervention (targeted therapy) and comparator group. Outcome events were presented so that an OR of more than 1 indicated a beneficial effect of the targeted therapy. Heterogeneity statistics were calculated according to a fixed-effect meta-analytic summary and presented as the apparent inconsistency between trials (I^2^) [21]. However, for the meta-regression analyses we used random-effects models as we, anticipated severe heterogeneity across studies. Effect estimates were calculated using the SAS procedure "Proc Mixed" that fits mixed linear models including variance component models [22]; we chose this approach rather than the DerSimonian and Laird estimator [23] because the latter is likely to produce biased estimates with falsely high precision [24].

According to our objective, we explored potential effect modification of trial eligibility criteria and patient baseline characteristics, in a number of meta-regression analyses. These analyses were modelled using Restricted Maximum Likelihood (REML)-based models. A priori, we defined a relevant study-level covariate as one that decreased the between-study variance, Tau^2^ (T^2^) as a consequence of inclusion in the (mixed effects) statistical model. Comparisons between different trial eligibility criteria or patient baseline characteristics are presented as the Ratio of Odds Ratios (ROR) with a 95% confidence interval (95%CI).[25] Analyses were performed using Review Manager for basic meta-analyses (Version 5.1. Copenhagen: The Nordic Cochrane Centre, The Cochrane Collaboration, 2008), and SAS software for the multivariable meta-regression models (version 9.3, by SAS Institute Inc., Cary, NC, USA).

## Results

**Fig 1:**
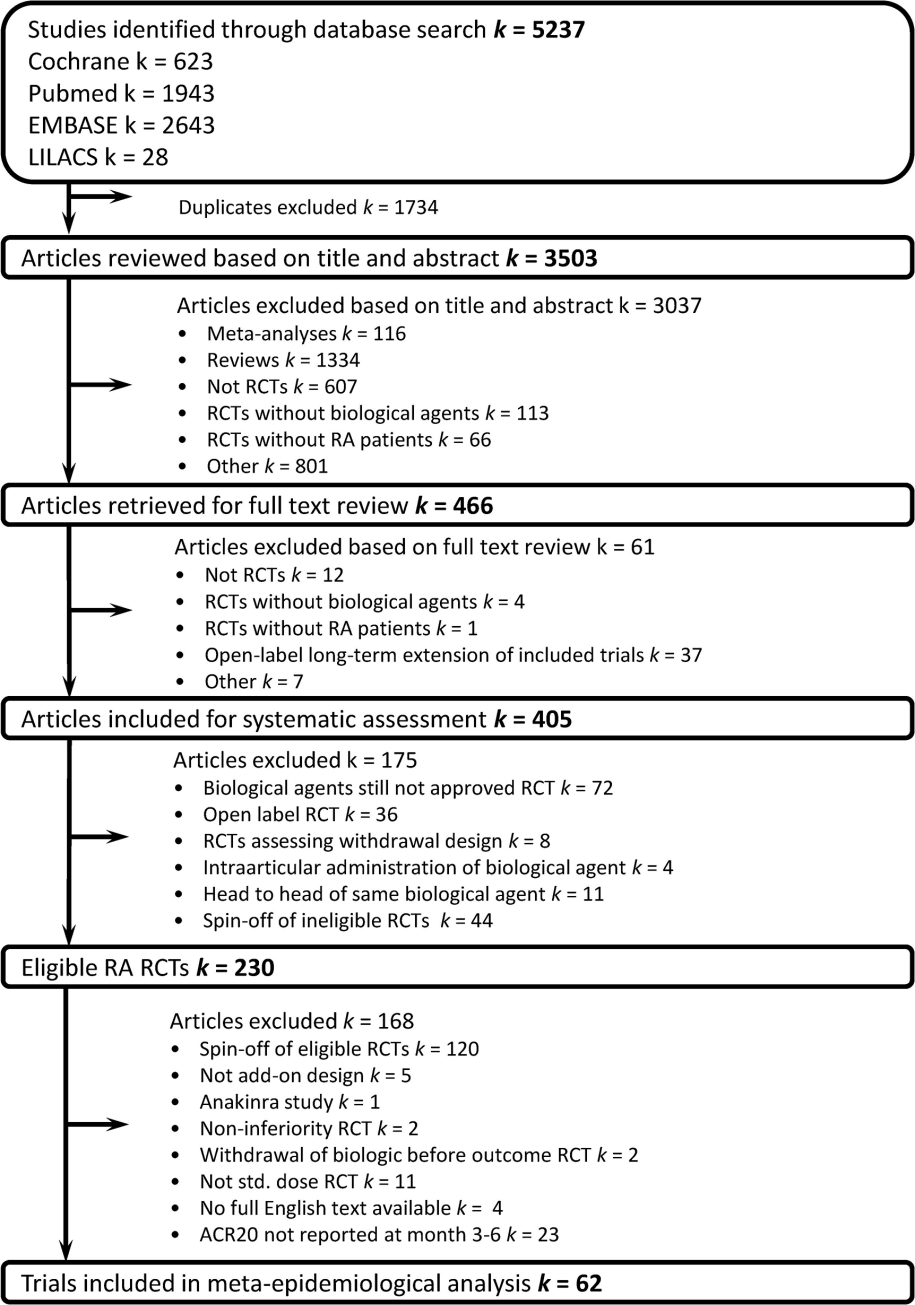
Flow diagram of included randomized controlled trials. RCT, randomized controlled trial; RA, rheumatoid arthritis.

As illustrated in **Fig 1**, from 230 possible RA studies, a total of 62 trials were considered eligible, including 19,923 RA patients in the primary analysis. Of these trials, 35 also reported data on DAS28-remission and were included for the secondary analyses. **(S1 Table)** presents the characteristics of the included trials, which were overall of a good methodological quality (low RoB).

There were only two missing values in the extraction of trial eligibility criteria; one study did not report a minimum required SJC and TJC at inclusion, which later was remedied by using datasets' median values [26]. Missing values concerning patient baseline characteristics; 17 trials did not report a baseline DAS28. We approximated this missing information by using the DAS28 formulas based on erythrocyte sedimentation rate (DAS28-ESR(4)) or CRP (DAS28-CRP(4)) to calculate the DAS28 baselines from available data. Data on the following patient baseline characteristics were not reported and thus were handled by imputation of the datasets' median value for that variable: female sex (1 trial, n missing = 20 patients), rheumatoid factor (16 trials, n missing = 4,494 patients), CRP (2 trials, n missing = 517 patients), SJC (2 trials, n missing = 193 patients), TJC (2 trials, n missing = 193 patients), disease duration (2 trials, n missing = 58 patients), MD global (20 trials, n missing = 5644 patients), patient global (18 trials, n missing = 6450 patients), patient pain (20 trials, n missing = 6422 patients), and health assessment questionnaire–disability index (11 trials, n missing = 2894). Baseline anti-CCP was reported in only 11 trials and was not considered further.

### Primary outcome

**Table 1**presents the analyses of trial eligibility criteria using ACR20 as the outcome. Corresponding analyses of patient baseline characteristics for ACR20 are seen in **Table 2**.

**Table 1:** Results of the stratified meta-analyses for trial eligibility criteria modifying ACR20 response.

Trial eligibility criteria					
Variable:	Trials	OR (95% CI)	CE Rate	τ^2^	p-interaction
Overall	62	3.96 (3.41 to 4.60)	0.27	0.25	N.A.
**DMARD History**				0.16	0.0002
DMARD-Naïve	8	1.97 (1.39 to 2.79)	0.55		
DMARD-IR	49	4.34 (3.75 to 5.01)	0.26		
TT-IR	5	3.92 (2.58 to 5.97)	0.18		
**csDMARD Handling at Randomisation**				0.24	0.22
Naive	1	6.00 (0.65 to 55.01)	0.20		
Not Using	18	3.22 (2.46 to 4.21)	0.31		
Continued	9	3.44 (2.43 to 4.87)	0.25		
Discontinued	33	4.57 (3.72 to 5.61)	0.24		
Not Reported	1	6.89 (2.16 to 22.02)	0.27		
**MTX Handling at Randomisation**				0.18	0.002
Naive	7	2.06 (1.39 to 3.04)	0.53		
Not Using	3	4.60 (2.55 to 8.30)	0.28		
Continued	42	3.95 (3.38 to 4.62)	0.27		
Discontinued	10	5.62 (3.95 to 7.99)	0.19		
Not Reported	0	N.A.	N.A.		
**TT Handling at Randomisation**				0.24	0.23
Naive	19	3.33 (2.55 to 4.34)	0.27		
Not Using	14	5.01 (3.72 to 6.76)	0.23		
Continued	0	N.A.	N.A.		
Discontinued	16	4.11 (3.06 to 5.51)	0.27		
Not Reported	13	3.67 (2.63 to 5.12)	0.35		
**Max Disease Duration at Inclusion**				0.22	0.051
Early Arthritis (≤2years)	5	2.29 (1.35 to 3.89)	0.53		
Not Reported	49	3.94 (3.36 to 4.61)	0.26		
Established Arthritis (>2 years)	8	5.18 (3.51 to 7.66)	0.28		
**Min Required CRP at Inclusion**				0.25	0.47
4.5- 7mg/L or more	10	3.03 (2.10 to 4.37)	0.26		
10 mg/L or more	18	4.09 (3.15 to 5.32)	0.26		
15 mg/L or more	12	3.93 (2.82 to 5.47)	0.31		
20 mg/L or more	11	4.98 (3.42 to 7.25)	0.20		
No Criteria Reported	11	3.94 (2.67 to 5.83)	0.31		
**Serology**				0.25	0.29
Mixed	58	4.05 (3.47 to 4.73)	0.26		
Only Seropositive	4	2.95 (1.68 to 5.19)	0.33		
Only Seronegative	0	N.A.	N.A.		
**Min Required 66 SJC at Inclusion**				0.22	0.01
≥3	1	1.21 (0.39 to 3.73)	0.49		
≥4	5	2.70 (1.68 to 4.33)	0.31		
≥6	31	4.13 (3.37 to 5.06)	0.26		
≥8	6	3.07 (1.99 to 4.73)	0.33		
≥9	5	7.40 (4.50 to 12.17)	0.14		
≥10	14	3.73 (2.75 to 5.05)	0.33		
**Min Required 68 TJC at Inclusion**				0.25	0.33
≥4	5	2.71 (1.65 to 4.45)	0.31		
≥6	17	4.32 (3.23 to 5.77)	0.25		
≥8	14	3.63 (2.67 to 4.94)	0.27		
≥9	10	5.22 (3.59 to 7.60)	0.26		
≥10	4	4.77 (2.51 to 9.06)	0.38		
≥12	12	3.54 (2.54 to 4.95)	0.27		

CE, control event; CRP, C-reactive protein; DAS28, disease activity score in 28 joints; csDMARD, conventional synthetic disease modifying antirheumatic drug; IR, inadequate responders; MTX, metothrexate; OR, odds ratio; RF, rheumatoid factor; SJC, swollen joint count; TJC, tender joint count; TT, Targeted therapy.

**Table 2:** Results of the stratified meta-analyses for patient baseline characteristics modifying response (ACR20 and DAS28-remission state).

**ACR20 response**				
**Variable**	**Trials**	**Coefficient (95% CI)**	τ^2^	**p-value**
BL Female (%)	62	1.02 (0.98 to 1.06)	0.25	0.31
BL Age (years)	62	1.02 (0.95 to 1.10)	0.26	0.56
BL RF (%)	62	1.01 (0.99 to 1.03)	0.26	0.48
BL DAS28	62	1.22 (0.86 to 1.73)	0.24	0.26
BL CRP (mg/mL)	62	1.01 (1.00 to 1.02)	0.25	0.23
BL 66 SJC	62	0.99 (0.95 to 1.04)	0.26	0.78
BL 68 TJC	62	0.98 (0.95 to 1.01)	0.26	0.13
BL Disease Duration (Years)	62	1.05 (1.00 to 1.11)	0.23	0.03
BL HAQ-DI	62	0.65 (0.30 to 1.41)	0.26	0.28
BL MD Global (0–100)	62	1.02 (0.98 to 1.05)	0.25	0.41
BL PT Global (0–100)	62	1.01 (1.00 to 1.03)	0.24	0.15
BL VAS_pain_ (0–100)	62	1.01 (0.99 to 1.03)	0.25	0.35
**DAS28-remission**				
**Variable**	**Trials**	**OR (95% CI)**	τ^2^	**P-value**
BL Female (%)	35	1.02 (0.95 to 1.11)	0.32	0.55
BL Age (years)	35	1.13 (0.96 to 1.33)	0.29	0.13
BL RF (%)	35	0.99 (0.94 to 1.03)	0.32	0.51
BL DAS28	35	1.59 (0.74 to 3.42)	0.29	0.23
BL CRP (mg/mL)	35	1.00 (0.96 to 1.03)	0.33	0.94
BL 66 SJC	35	1.01 (0.93 to 1.10)	0.33	0.73
BL 68 TJC	35	1.01 (0.97 to 1.06)	0.32	0.56
BL Disease Duration (Years)	35	1.11 (1.03 to 1.20)	0.17	0.005
BL HAQ-DI	35	0.73 (0.17 to 3.14)	0.33	0.67
BL MD Global (0–100)	35	1.00 (0.91 to 1.09)	0.33	0.93
BL PT Global (0–100)	35	1.01 (0.99 to 1.03)	0.33	0.39
BL VAS_pain_ (0–100)	35	1.01 (0.98 to 1.03)	0.34	0.50

BL, baseline; CRP, C-reactive protein; DAS28, disease activity score in 28 joints; HAQ-DI, health assessment questionnaire–disability index;MD Global, physicians global assessment (0–100); PT Global, patient global assessment (0–100); RF, rheumatoid factor; SJC, swollen joint count; TJC, tender joint count; VAS_pain_, visual analogue scale for pain (0–100).

**Fig 2:**
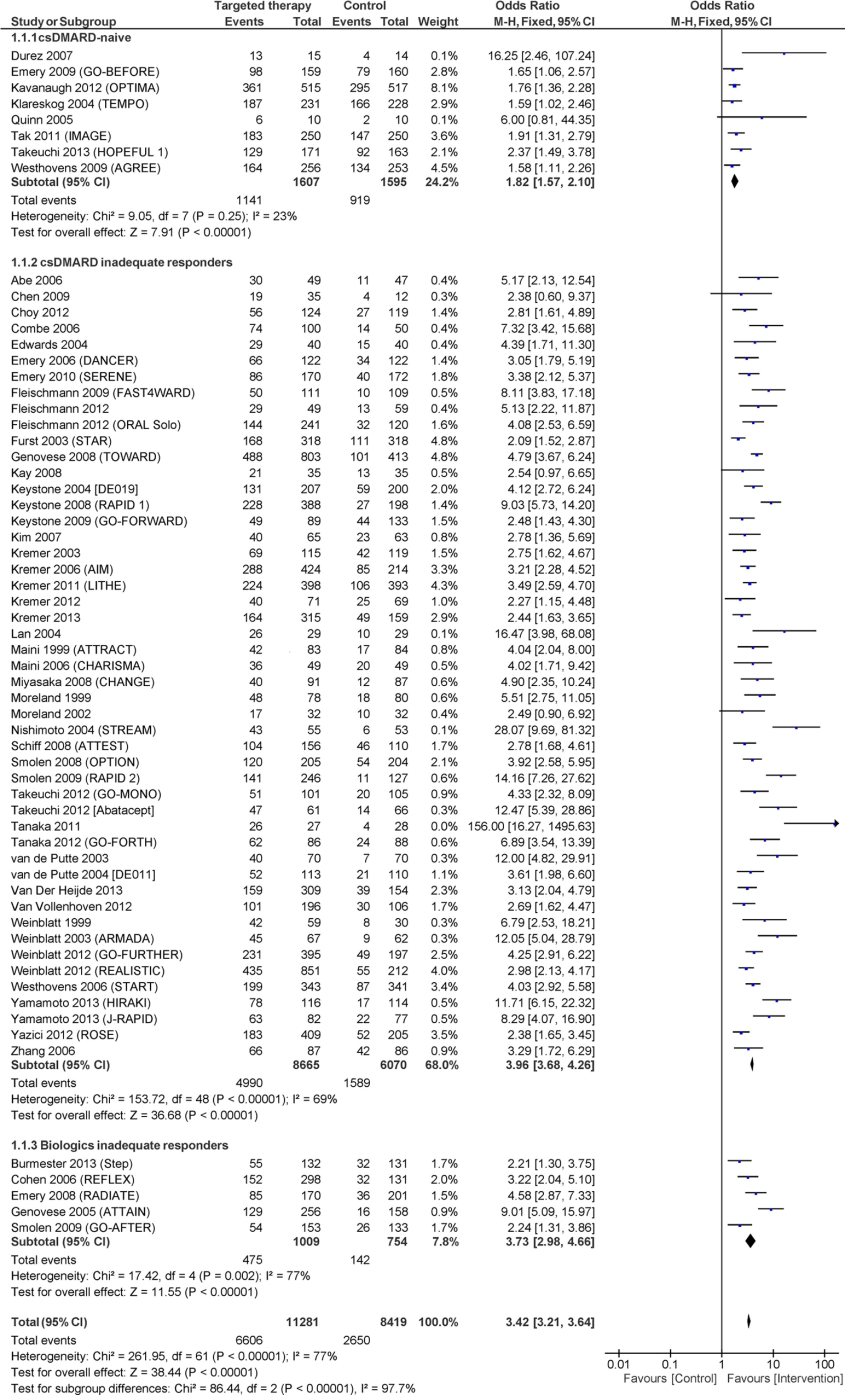
Forest plot showing the effect (ACR20 response) of included trials, stratified by the included patients DMARD-history.

The overall effect-increase of adding a targeted therapy was OR 3.96 (95% CI 3.41 to 4.60). Between-study variance in trials (τ^2^) was 0.25, and the proportion of variance attributable to heterogeniety (I^2^) was 77%. The trial eligibility criterion "DMARD History" which grouped trials according to whether their patients were DMARD-naïve, DMARD-IR, or TT-IR, reduced the between-trial heterogeneity by 36% (τ^2^ = 0.16) with statistically significant interaction among the subgroups (p = 0.0002). The added benefit of targeted therapies was lower in DMARD-naïve trials with a lower ROR compared with DMARD-IR (0.45; 95% CI 0.31 to 0.66) and TT-IR (0.50; 95% CI 0.29 to 0.87) trials. **Fig 2**shows a forest plot of trials stratified by DMARD History.

The trial eligibility criterion "Maximum Disease Duration at Inclusion" did not show an overall statistically significant interaction among subgroups (p = 0.051). However, trials that only included patients with early arthritis had significantly lower effect compared with trials that only included patients with established arthritis (ROR 0.44; 95% CI 0.23 to 0.85, p = 0.02). The patient baseline characteristic "Baseline Disease Duration" demonstrated increasing effect with longer average disease duration of the trial patients (β = 1.05, 1.00 to 1.11, p = 0.03) with 1.05 OR-units per year.

The trial eligibility criteria "MTX Handling at Randomization" and "Minimum Required SJC at Inclusion" also demonstrated significant interactions among subgroups (p = 0.002 and p = 0.01, respectively).

The median absolute response rates for each “DMARD History” group are shown in S1 Fig.

### Secondary outcome

**Table 3**presents the analyses conducted using DAS28-remission as the outcome. Corresponding analyses of patient baseline characteristics for DAS28-remission are seen in **Table 2**.

**Table 3:** Results of the stratified meta-analyses for trial eligibility criteria modifying DAS28-remission.

Trial eligibility criteria					
VARIABLE:	Trials	OR (95% CI)	CE Rate	τ^2^	p-interaction
Overall	35	5.57 (4.24 to 7.3)	0.026	0.32	N.A.
**DMARD History**				0.16	0.003
DMARD-Naive	5	2.93 (1.93 to 4.46)	0.135		
DMARD-IR	25	6.27 (4.71 to 8.34)	0.026		
TT-IR	5	10.30 (4.61 to 23.01)	0.016		
**csDMARD Handling at Randomisation**				0.29	0.34
Naive	0	N.A.	N.A.		
Not Using	10	4.01 (2.45 to 6.55)	0.015		
Continued	8	6.73 (3.99 to 11.32)	0.020		
Discontinued	16	5.75 (3.85 to 8.59)	0.028		
Not Reported	1	13.42 (2.65 to 67.99)	0.034		
**MTX Handling at Randomisation**				0.18	0.007
Naive	4	2.87 (1.76 to 4.66)	0.13		
Not Using	2	4.43 (1.82 to 10.81)	0.072		
Continued	26	7.10 (5.31 to 9.50)	0.018		
Discontinued	3	2.86 (1.14 to 7.17)	0.034		
Not Reported	0	N.A.	N.A.		
**TT Handling at Randomisation**				0.15	0.006
Naive	10	3.48 (2.46 to 4.94)	0.072		
Not Using	11	6.46 (4.14 to 10.06)	0.016		
Continued	0	N.A.	N.A.		
Discontinued	11	8.95 (5.72 to 14.00)	0.017		
Not Reported	3	3.96 (2.04 to 7.71)	0.028		
**Max Disease Duration at Inclusion**				0.27	0.09
Early Arthritis (≤2years)	3	2.83 (1.48 to 5.40)	0.147		
Not Reported	28	6.19 (4.59 to 8.33)	0.025		
Established Arthritis (>2 years)	4	6.51 (2.69 to 15.78)	0.008		
**Min Required CRP at Inclusion**				0.23	0.04
4.5- 7mg/L or more	9	3.15 (1.89 to 5.24)	0.026		
10 mg/L or more	12	8.82 (5.79 to 13.44)	0.028		
15 mg/L or more	9	5.04 (3.12 to 8.13)	0.020		
20 mg/L or more	3	5.10 (2.19 to 11.86)	0.021		
No Criteria Reported	2	4.09 (1.63 to 10.25)	0.074		
**Serology**				0.30	0.17
Mixed	34	5.79 (4.40 to 7.62)	0.025		
Only Seropositive	1	2.41 (0.71 to 8.18)	0.079		
Only Seronegative	0	N.A.	N.A.		
**Min Required 66 SJC at Inclusion**				0.38	0.95
≥3	0	N.A.	N.A.		
≥4	5	6.13 (2.85 to 13.20)	0.026		
≥6	17	5.96 (3.95 to 9.00)	0.021		
≥8	3	4.66 (1.95 to 11.10)	0.026		
≥9	3	8.07 (2.07 to 31.46)	0.008		
≥10	7	4.95 (2.67 to 9.17)	0.028		
**Min Required 66 TJC at Inclusion**				0.39	0.91
≥4	5	6.14 (2.84 to 13.28)	0.026		
≥6	11	5.21 (2.96 to 9.15)	0.017		
≥8	8	6.87 (4.01 to 11.76)	0.030		
≥9	3	8.08 (2.07 to 31.63)	0.008		
≥10	2	4.08 (1.42 to 11.67)	0.114		
≥12	6	4.75 (2.44 to 9.26)	0.028		

CE, control event; CRP, C-reactive protein; DAS28, disease activity score in 28 joints; csDMARD, conventional synthetic disease modifying antirheumatic drug; IR, inadequate responders; MTX, metothrexate; OR, odds ratio; RF, rheumatoid factor; SJC, swollen joint count; TJC, tender joint count; TT, Targeted therapy.

**Fig 3:**
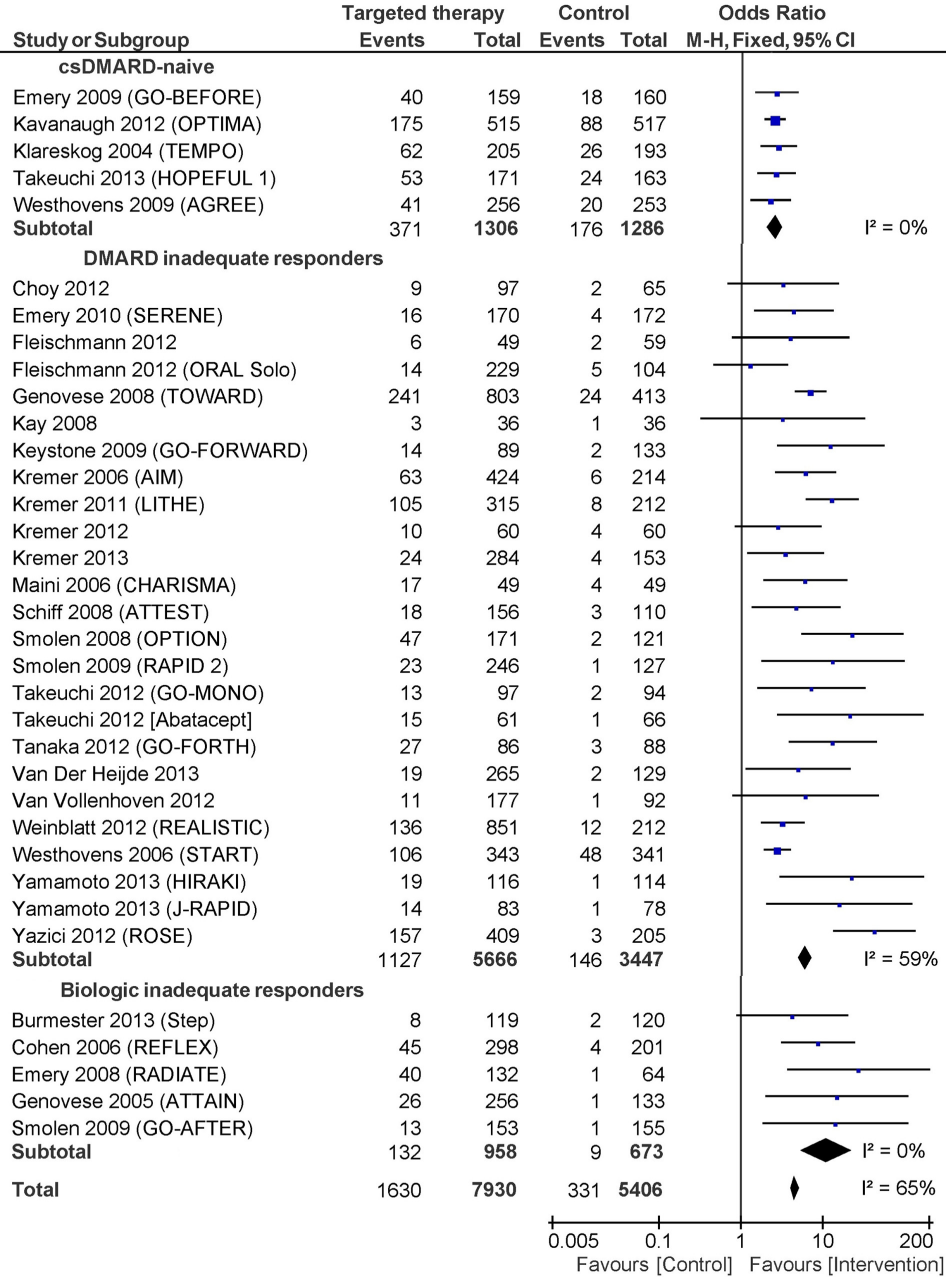
Forest plot showing the effect (DAS28-remission) of included trials, stratified by the included patients DMARD-history.

The overall effect-increase of adding a targeted therapy was OR 5.57 (95% CI 4.24, 7.30). Between-study variance in trials (τ^2^) was 0.32, and the proportion of variance attributable to heterogeniety (I^2^) was 65%. The trial eligibility criterion "DMARD History" reduced the between-trial heterogeneity by 50% (τ^2^ = 0.16) with statistically significant interaction among the subgroups (p = 0.003). The added benefit of targeted therapies was lower in DMARD-naïve trials with a lower likelihood of responding compared with DMARD-IR (ROR = 0.47; 95% CI 0.28 to 0.78) and TT-IR trials (ROR = 0.28; 95% CI 0.11 to 0.70). **Fig 3**shows a forest plot of trials stratified by DMARD History.

There was no statistical significant interaction among subgroups for the trial eligibility criterion "Maximum Disease Duration at Inclusion" (p = 0.09), whereas the patient baseline characteristic "Baseline Disease Duration" demonstrated increasing effect with longer average disease duration of the trial patients (p = 0.005).

The trial eligibility criteria "MTX Handling at Randomization," "TT Handling at Randomization," and "Minimum Required CRP at Inclusion" also demonstrated significant interactions among subgroups (p = 0.007, p = 0.006, and p = 0.04, respectively).

### Ancillary analyses

To explore the confounding effect of the trial eligibility criterion "DMARD History" in the remaining trial eligibility criteria and patient baseline characteristics, we omitted trials labelled as DMARD-naïve when conducting post-hoc analyses. Results from these modified datasets (ACR20-Modified, DAS28-remission-Modified) are shown in (**S2 and S3 Tables**). In the ACR20-Modified dataset; "Minimum Required 66 SJC at Inclusion" no longer demonstrated significant interaction among subgroups, which suggests confounding by the included patients' DMARD history. "MTX Handling at Randomization" still showed significant interaction among subgroups (p = 0.02), but inspection of the trials that discontinued MTX showed that the control group received no treatment (these were primarily monotherapy and dose-finding studies). Trials solely including patients with established arthritis had greater added effect of a targeted therapy compared to trials where “Max Disease Duration at Inclusion” was not specified or reported (ROR = 2.22; 95% CI 1.49 to 3.29). The patient baseline characteristic “Baseline RF (%)” showed increasing effect with greater proportion of RF positive patients at baseline (β = 1.02 (95% CI 1.00 to 1.04; p = 0.03)) with 1.02 OR-units per percentage point increase.

In the DAS28-remission dataset; the trial eligibility criterion "Minimum Required CRP at Inclusion" significantly modified effect, but there was no trend for increasing effect with a higher required CRP. However, in the DAS28-remission-Modified dataset, the CRP requirement at trial inclusion significantly modified effect and showed increasing effect with greater CRP requirement. Both "MTX Handling at Randomization" and "TT Handling at Randomization" no longer showed significant interaction, suggesting confounding by the included patients' DMARD history.

## Discussion

We analyzed a dataset including 62 trials representing a wide range of rheumatoid arthritis patients, who were allocated to one of nine used targeted therapies. RCTs testing various therapies have typically used trial eligibility criteria to select patients with the highest disease activity. Concerns have been voiced over the minority of RA patients in routine care who are eligible candidates for typical clinical trials testing new drugs [27]. In our study, most of the trial eligibility criteria did not modify overall treatment effect. In other words, trials with a highly selective inclusion do not show better effect of a targeted therapy as might otherwise be expected. Therefore, a selective inclusion might also not be necessary when designing trials to test new bDMARDs or tsDMARDs.

Our study also shows that the *added* effect of targeted therapies is only half the size in trials including DMARD-naïve patients, compared with trials including patients with an inadequate response to previous DMARD or targeted therapy. It has recently been suggested to start temporary treatment with a biological agent combined with MTX in newly diagnosed RA patients to take advantage of a potential window of opportunity [28;29]. Although such a strategy might yield better absolute response rates, our data show that the added effect of biological agents is less potent in this setting.

We found that absolute response rates differed for the control arms across the three “DMARD History” groups, which was expected (**S1 Fig**). Trials including DMARD-IR of TT-IR patients randomize patients to a control treatment that they have previously failed on which results in a large relative effect of the added active treatment and hence a larger odds ratio. However, we found that the added benefit of a targeted therapy was lower even when looking at absolute response rates for trials including DMARD-naïve patients–thus demonstrating the diminished effect (**S1 Fig**). We believe that these findings justify that all RA patients should be exposed to csDMARD treatment before introduction a targeted therapy is considered.

Both "Maximum Disease Duration at Inclusion" and "Baseline Disease Duration" reflect the same construct and our analyses support previous findings of greater added effect of targeted therapies in patients with longer disease duration [5;7]. However, when removing all DMARD-naïve trials from the dataset (**S2 Table**), trials solely including patients with established arthritis had greater added effect of a targeted therapy compared to trials where maximum disease duration at inclusion was not specified (i.e. patients were not selected on the basis of their disease duration). On the other hand, the patient baseline characteristic “Baseline Disease Duration” was no longer statistically significant, which suggests confounding by the “DMARD-History” (i.e. trials including DMARD-naxve participants also had the shortest disease durations) (**S2 Table**). As these two variables (both reflecting disease duration) give different results in the ancillary analyses we are unable to conclude whether disease duration is an independent effect modifier or whether it is confounded by the fact that DMARD-naïve trials also included patients with the shortest disease duration.

All trial eligibility criteria and patient baseline characteristics were analyzed using both ACR20 and DAS28-remission as the outcome of interest. Although these two outcomes are very different in nature, the results for “DMARD History” and disease duration were very similar for both outcomes. Some trial eligibility criteria were significant in the primary or secondary analyses, but not in the ancillary analyses. They were believed to be confounded by the trial characteristic “DMARD History” and not considered further (see Ancillary analyses for further details).

One difference between the primary/secondary analyses and the ancillary analyses deserves special attention. "Minimum Required CRP at Inclusion" modified the effect in the secondary analysis (DAS28-remission), but not in the primary analysis (ACR20). In the DAS28-remission-Modified dataset (trials categorized as “DMARD-naïve” were omitted in the modified datasets), this trial eligibility criterion showed significant interaction among subgroups and it was borderline significant in the ACR20-Modified dataset. This indicates that baseline CRP had different effects in different sub-groups; it could indicate a positive prognostic value of patients' having an elevated baseline CRP when looking solely at patients who have failed previous treatment with csDMARDs and/or bDMARDs/ tsDMARDs.

### Strengths and weaknesses

This study is the most comprehensive analysis to date of the effect of csDMARDs and bDMARDs/ tsDMARDs for RA. Only FDA/EMA-recommended dosages were included, which adds to the external validity of the study. Previous meta-epidemiological studies in this field have focused mainly on patient baseline characteristics whereas we also looked into the trial eligibility criteria. We focused on short-term (3–6 months) follow-up, which is also the time frame where it is relevant for a primary evaluation of the initiated treatments efficacy.

This study has several limitations. All extracted variables were limited to publicly available information. Outcome data were limited to clinical endpoints (i.e., no data on structural joint damage). Missing data could limit our findings for some variables, since this was handled by imputing the median value for that variable (i.e. the median of all the reported values for that specific variable). This conservative approach could mean overlooking potential effect modifiers concerning variables with many missing data Finally, all data were obtained at trial level; use of aggregated data to draw conclusions on individuals should be done with care. Because confounders and effect modification cannot be controlled with aggregated data, using aggregated data can give rise to wrongful conclusions—a phenomenon known as ecological fallacy [30]. It is therefore recommended that these findings be confirmed using individual patient data before any conclusions are drawn regarding their prognostic value.

## Conclusions

We have demonstrated that the majority of trial eligibility criteria and patient baseline characteristics cannot be considered effect modifiers in trials testing a targeted therapy for rheumatoid arthritis. This suggests that trials with a highly selective inclusion do not show better effect of a targeted therapy as might otherwise be expected. Therefore, a selective inclusion might also not be necessary when designing trials to test new bDMARDs or tsDMARDs.

We found that the medication history and baseline disease duration of the included participants modified the added effect of the targeted therapy. The added benefit of a bDMARD or tsDMARD was lower in trials including patients who were DMARD-naïve.

Researchers conducting meta-analyses, assessing the efficacy of targeted therapies for RA, should include the patients medication history as a covariate in order to improve the precision of estimates. We recommended that future trials report adequately on the patients previously failed number of csDMARDs and bDMARDs/ tsDMARDs. Also the trial definition of treatment failure for each class of drugs should be given.

### Data Sharing

The dataset has been published as supporting information (**S4 Table**).

## Supporting Information

S1 Fig Absolute response rates by DMARD-history. The figure shows the median absolute response rate for each DMARD-history group. The figure visualizes the response in the control arm (black) and shows how much added effect there was in the arm receiving the targeted therapy (grey).
(TIF)

S1 Table Overview of included trials (DOCX)

S2 Table ACR20 data omitting DMARD-naïve trials (DOCX)

S3 Table DAS28-remission data omitting DMARD-naïve trials (DOCX)

S4 Table Full dataset (PDF)
