# Supplementary figures and images for: Most Trial Eligibility Criteria and Patient Baseline Characteristics Do Not Modify Treatment Effect in Trials Using Targeted Therapies for Rheumatoid Arthritis: A Meta-Epidemiological Study
Source: PLoS One. 2015 Sep 11;10(9):e0136982. doi: 10.1371/journal.pone.0136982 (PMC4567072; doi:10.1371/journal.pone.0136982)

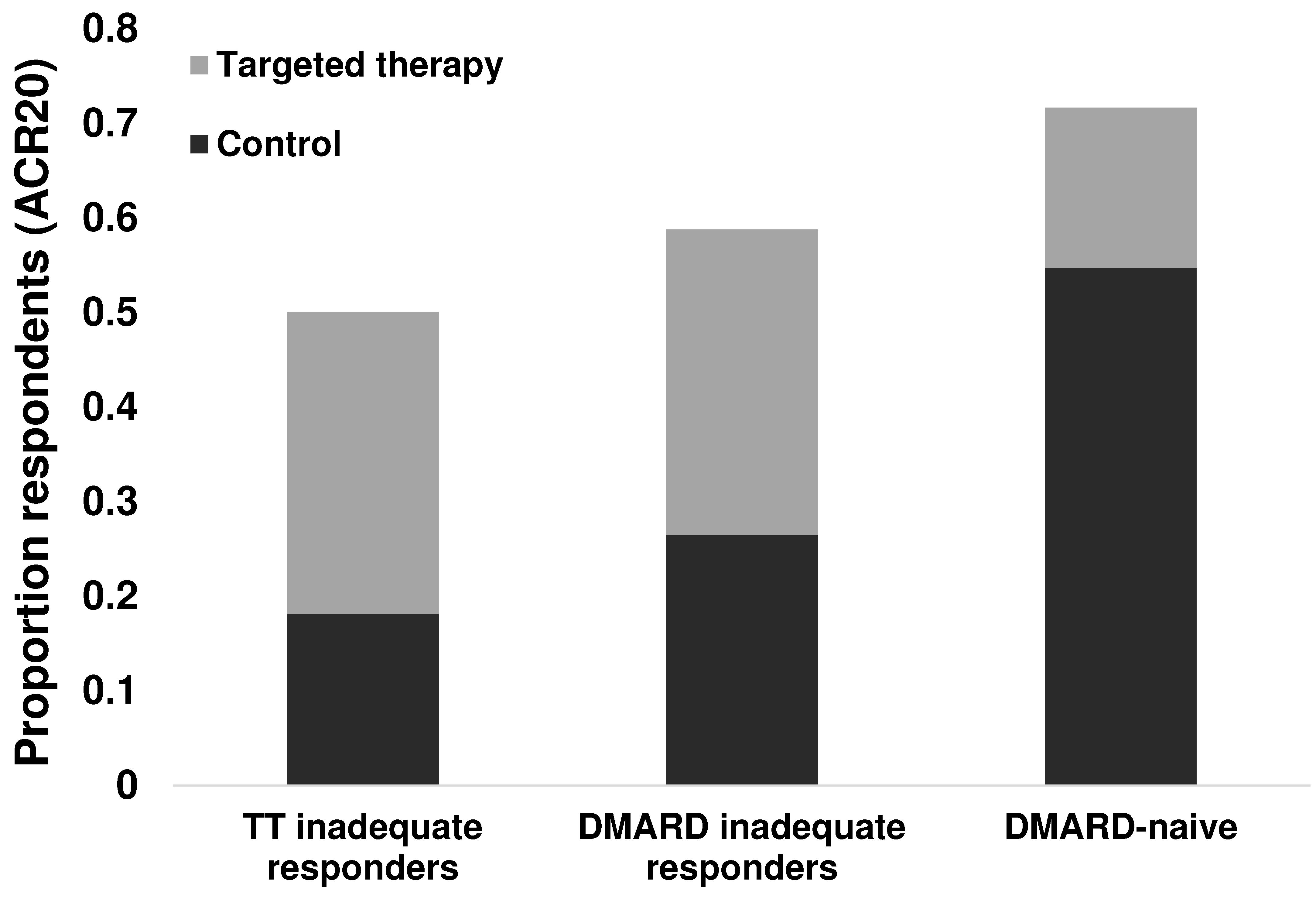

Supplement: S1 Fig — The figure shows the median absolute response rate for each DMARD-history group. The figure visualizes the response in the control arm (black) and shows how much added effect there was in the arm receiving the targeted therapy (grey). (TIF) [file pone.0136982.s001.tif]
