# Supplementary material for: Most Trial Eligibility Criteria and Patient Baseline Characteristics Do Not Modify Treatment Effect in Trials Using Targeted Therapies for Rheumatoid Arthritis: A Meta-Epidemiological Study
Source: PLoS One. 2015 Sep 11;10(9):e0136982. doi: 10.1371/journal.pone.0136982 (PMC4567072; doi:10.1371/journal.pone.0136982)
Supplement: S1 Table — (DOCX) [file pone.0136982.s002.docx]

**S1 - Supplementary table 1**. Overview of included randomised trials

| **Trial** | **Arm** | **n** | **DAS28 remission reported** | **Extracted outcome week** |
| --- | --- | --- | --- | --- |
| Maini et al.  (1999) | INF 3 mg/kg + MTX  PLA + MTX | 86  88 | No | 30 |
| Weinblatt et al.  (1999) | ETA 25 mg + MTX  PLA + MTX | 59  30 | No | 24 |
| Moreland et al.  (1999) | ETA 25 mg  PLA | 81  83 | No | 13 |
| Moreland et al.  (2002) | ABA 10mg/kg  PLA | 32  32 | No | 12 |
| Kremer et al.  (2003) | ABA 10mg/kg + MTX  PLA + MTX | 115  119 | No | 26 |
| Furst et al.  (2003) | ADA 40 mg  PLA | 318  318 | No | 24 |
| Weinblatt et al.  (2003) | ADA 40 mg + MTX  PLA + MTX | 67  62 | No | 24 |
| van de Putte et al.  (2003) | ADA 40 mg  PLA | 70  70 | No | 12 |
| Edwards et al.  (2004) | RIT 1000 mg + MTX  PLA + MTX | 40  40 | No | 24 |
| Klareskog et al.  (2004) | ETA 25mg + MTX  PLA + MTX | 229  229 | Yes | 24 |
| Keystone et al.  (2004) | ADA 40 mg + MTX  PLA + MTX | 207  200 | No | 24 |
| van de Putte et al.  (2004) | ADA 40 mg  PLA | 113  110 | No | 26 |
| Nishimoto et al.  (2004) | TOC 8 mg/kg  PLA | 55  54 | No | 12 |
| Lan et al.  (2004) | ETA 25 mg + MTX  PLA + MTX | 29  29 | No | 12 |
| Genovese et al.  (2005) | ABA 10 mg/kg  PLA | 262  131 | Yes | 24 |
| Quinn et al.  (2005) | INF 3 mg/kg + MTX  PLA + MTX | 10  10 | No | 14 |
| Kremer et al.  (2006) | ABA 10 mg/kg + MTX  PLA + MTX | 433  219 | Yes | 26 |
| Maini et al.  (2006) | TOC 8 mg/kg + MTX  PLA + MTX | 50  49 | Yes | 16 |
| Cohen et al.  (2006) | RIT 1000 mg + MTX  PLA + MTX | 311  209 | Yes | 24 |
| Emery et al.  (2006) | RIT 1000 mg + MTX  PLA + MTX | 128  128 | No | 24 |
| Combe et al.  (2006) | ETA 25 mg + SSZ  PLA + SSZ | 104  52 | No | 24 |
| Abe et al.  (2006) | INF 3 mg/kg + MTX  PLA + MTX | 50  50 | No | 14 |
| Westhovens et al.  (2006) | INF 3 mg/kg + MTX  PLA + MTX | 360  363 | Yes | 22 |
| Zhang et al.  (2006) | INF 3 mg/kg + MTX  PLA + MTX | 87  86 | No | 18 |
| Kim et al.  (2007) | ADA 40 mg + MTX  PLA + MTX | 65  63 | No | 24 |
| Durez et al.  (2007) | INF 3 mg/kg + MTX  PLA + MTX | 15  14 | No | 22 |
| Genovese et al.  (2008) | TOC 8 mg/kg  PLA | 805  415 | Yes | 24 |
| Smolen et al.  (2008) | TOC 8 mg/kg + MTX  PLA + MTX | 205  204 | Yes | 24 |
| Schiff et al.  (2008) | ABA 10 mg/kg + MTX  PLA + MTX | 156  110 | Yes | 28 |
| Emery et al.  (2008) | TOC 8 mg/kg + MTX  PLA + MTX | 175  160 | Yes | 24 |
| Miyasaka et al.  (2008) | ADA 40 mg  PLA | 91  87 | No | 24 |
| Kay et al.  (2008) | GOL 50 mg + MTX  PLA + MTX | 35  35 | Yes | 16 |
| Keystone et al.  (2008) | CER 200mg + MTX  PLA + MTX | 393  199 | No | 24 |
| Chen et al.  (2009) | ADA 40 mg + MTX  PLA + MTX | 35  12 | No | 12 |
| Emery et al.  (2009) | GOL 50 mg + MTX  PLA + MTX | 159  160 | Yes | 24 |
| Keystone et al.  (2009) | GOL 50 mg + MTX  PLA + MTX | 89  133 | Yes | 14 |
| Smolen et al.  (2009) | GOL 50 mg  PLA | 153  155 | Yes | 14 |
| Fleischmann et al.  (2009) | CER 400 mg  PLA | 111  109 | No | 24 |
| Smolen et al.  (2009) | CER 200 mg + MTX  PLA + MTX | 246  127 | Yes | 24 |
| Westhovens et al.  (2009) | ABA 10 mg + MTX  PLA + MTX | 256  255 | Yes | 12 |
| Emery et al.  (2010) | RIT 1000 mg + MTX  PLA + MTX | 172  172 | Yes | 24 |
| Kremer et al.  (2011) | TOC 8 mg/kg + MTX  PLA + MTX | 399  392 | Yes | 24 |
| Tak et al.  (2011) | RIT 1000 mg + MTX  PLA + MTX | 251  252 | No | 24 |
| Tanaka et al.  (2011) | TOF 5 mg + MTX  PLA + MTX | 28  28 | No | 12 |
| Yazici et al.  (2012) | TOC 8 mg/kg  PLA | 412  207 | Yes | 24 |
| Takeuchi et al.  (2012) | GOL 50 mg  PLA | 102  110 | Yes | 14 |
| Fleischmann et al.  (2012) | TOF 5 mg  PLA | 244  122 | Yes | 13 |
| Fleischmann et al.  (2012) | TOF 5 mg  PLA | 50  59 | Yes | 12 |
| Choy et al.  (2012) | CER 400 mg + MTX  PLA + MTX | 126  121 | Yes | 24 |
| Kavanaugh et al.  (2012) | ADA 40 mg + MTX  PLA + MTX | 515  517 | Yes | 26 |
| Takeuchi et al.  (2012) | ABA 10 mg/kg + MTX  PLA + MTX | 62  66 | Yes | 24 |
| Tanaka et al.  (2012) | GOL 50 mg + MTX  PLA + MTX | 89  90 | Yes | 14 |
| van Vollenhoven et al.  (2012) | TOF 5 mg + MTX  PLA + MTX | 204  108 | Yes | 26 |
| Weinblatt et al.  (2012) | GOL 2mg/kg + MTX  PLA + MTX | 395  197 | No | 14 |
| Weinblatt et al.  (2012) | CER 200 mg  PLA | 851  212 | Yes | 12 |
| Kremer et al.  (2012) | TOF 5 mg + MTX  PLA + MTX | 71  69 | Yes | 12 |
| Yamamoto et al.  (2013) | CER 200 mg  PLA | 116  114 | Yes | 12 |
| Yamamoto et al.  (2013) | CER 200 mg + MTX  PLA + MTX | 82  77 | Yes | 12 |
| van der Heijde et al.  (2013) | TOF 5 mg + MTX  PLA + MTX | 321  160 | Yes | 26 |
| Burmester et al.  (2013) | TOF 5 mg + MTX  PLA + MTX | 133  132 | Yes | 13 |
| Takeuchi et al.  (2013) | ADA 40 mg + MTX  PLA + MTX | 171  163 | Yes | 26 |
| Kremer et al.  (2013) | TOF 5 mg  PLA | 318  159 | Yes | 26 |

ABA, abatacept; ADA, adalimumab; Bio, biological agent; CER, certolizumab; DAS28, disease activity score in 28 joints; DMARD, disease modifying antirheumatic drugs; ETA, etanercept; GOL, golimumab; INF, infliximab; IR, insufficient responders; MTX, metothrexate; PLA, placebo; RIT, rituximab; SSZ, sulfasalazine; TOC, tocilizumab; TOF, tofacitinib.
