# Supplementary material for: Most Trial Eligibility Criteria and Patient Baseline Characteristics Do Not Modify Treatment Effect in Trials Using Targeted Therapies for Rheumatoid Arthritis: A Meta-Epidemiological Study
Source: PLoS One. 2015 Sep 11;10(9):e0136982. doi: 10.1371/journal.pone.0136982 (PMC4567072; doi:10.1371/journal.pone.0136982)
Supplement: S2 Table — (DOCX) [file pone.0136982.s003.docx]

**S2 – Supplementary table 2**

**ACR20 analysis, omitting all ‘DMARD-naïve’ trials (ACR20-Modified)**

| **Trial eligibility criteria** | | | | | | |
| --- | --- | --- | --- | --- | --- | --- |
| **VARIABLE:** | | **Trials** | **OR (95% CI)** | | τ^2^ | **p-interaction** |
| Overall | | 54 | 4.31 (3.74 to 4.98) | | 0.18 | N.A. |
| **DMARD History** | |  |  | | 0.19 | 0.65 |
| DMARD-Naïve | | 0 | N.A. | |  |  |
| DMARD-IR | | 49 | \| 4.37(3.75 to 5.09) \| \| --- \| | |  |  |
| TT-IR | | 5 | \| 3.93 (2.51 to 6.13) \| \| --- \| | |  |  |
| **csDMARD Handling at Randomisation** | |  |  | | 0.18 | 0.34 |
| Naive | | 0 | N.A. | |  |  |
| Not Using | | 12 | \| 4.34 (3.19 to 5.91) \| \| --- \| | |  |  |
| Continued | | 9 | \| 3.43 (2.51 to 4.67) \| \| --- \| | |  |  |
| Discontinued | | 32 | \| 4.62 (3.82 to 5.60) \| \| --- \| | |  |  |
| Not Reported | | 12 | \| 6.89 (2.37 to 20.02) \| \| --- \| | |  |  |
| **MTX Handling at Randomisation** | |  |  | | 0.14 | 0.02 |
| Naive | | 0 | N.A. | |  |  |
| Not Using | | 2 | \| 9.43 (4.58 to 19.42) \| \| --- \| | |  |  |
| Continued | | 42 | \| 3.93 (3.39 to 4.55) \| \| --- \| | |  |  |
| Discontinued | | 10 | \| 5.58 (4.00 to 7.80) \| \| --- \| | |  |  |
| Not Reported | | 0 | N.A. | |  |  |
| **TT Handling at Randomisation** | |  |  | | 0.19 | 0.72 |
| Naive | | 14 | \| 4.07 (3.05 to 5.42) \| \| --- \| | |  |  |
| Not Using | | 14 | \| 4.97 (3.77 to 6.54) \| \| --- \| | |  |  |
| Continued | | 0 | N.A. | |  |  |
| Discontinued | | 16 | \| 4.05 (3.09 to 5.31) \| \| --- \| | |  |  |
| Not Reported | | 10 | \| 4.22 (2.97 to 5.99) \| \| --- \| | |  |  |
| **Max Disease Duration at Inclusion** | |  |  | | 0.10 | 0.00007 |
| Early Arthritis (≤2years) | | 0 | N.A. | |  |  |
| Not Reported | | 48 | \| 3.88 (3.44 to 4.39) \| \| --- \| | |  |  |
| Established Arthritis (>2 years) | | 6 | \| 8.60 (5.92 to 12.50) \| \| --- \| | |  |  |
| **Min CRP at Inclusion** | |  |  | | 0.14 | 0.06 |
| 4.5- 7mg/L or more | | 9 | \| 3.21 (2.34 to 4.40) \| \| --- \| | |  |  |
| 10 mg/L or more | | 17 | \| 4.19 (3.37 to 5.20) \| \| --- \| | |  |  |
| 15 mg/L or more | | 9 | \| 5.33 (3.86 to 7.37) \| \| --- \| | |  |  |
| 20 mg/L or more | | 9 | \| 5.79 (4.11 to 8.15) \| \| --- \| | |  |  |
| No Criteria Reported | | 10 | \| 3.63 (2.61 to 5.04) \| \| --- \| | |  |  |
| **Serology** | |  |  | | 0.19 | 0.67 |
| Mixed | | 51 | \| 4.35 (3.75 to 5.05) \| \| --- \| | |  |  |
| Only Seropositive | | 3 | \| 3.80 (2.08 to 6.94) \| \| --- \| | |  |  |
| Only Seronegative | | 0 | N.A. | |  |  |
| **Min required 66 SJC at Inclusion** | |  |  | | 0.16 | 0.15 |
| ≥3 | | 1 | \| 3.29 (1.19 to 9.11) \| \| --- \| | |  |  |
| ≥4 | | 4 | \| 3.07 (1.91 to 4.93) \| \| --- \| | |  |  |
| ≥6 | | 28 | \| 4.16 (3.45 to 5.03) \| \| --- \| | |  |  |
| ≥8 | | 4 | \| 3.80 (2.34 to 6.17) \| \| --- \| | |  |  |
| ≥9 | | 5 | \| 7.38 (4.71 to 11.55) \| \| --- \| | |  |  |
| ≥10 | | 12 | \| 4.45 (3.28 to 6.04) \| \| --- \| | |  |  |
| **Min required 68 TJC at Inclusion** | |  |  | | 0.19 | 0.47 |
| ≥4 | | 4 | \| 3.08 (1.86 to 5.09) \| \| --- \| | |  |  |
| ≥6 | | 17 | \| 4.24 (3.26 to 5.52) \| \| --- \| | |  |  |
| ≥8 | | 10 | \| 4.02 (2.95 to 5.48) \| \| --- \| | |  |  |
| ≥9 | | 10 | \| 5.18 (3.69 to 7.28) \| \| --- \| | |  |  |
| ≥10 | | 3 | \| 6.86 (3.27 to 14.39) \| \| --- \| | |  |  |
| ≥12 | | 10 | \| 4.29 (3.06 to 6.02) \| \| --- \| | |  |  |
| **Patient baseline characteristics** | | | | | | |
| **VARIABLE:** | **Trials** | **Coefficient (95% CI)** | | τ^2^ | | **p-value** |
| BL Female (%) | 62 | \| 1.02 (0.98 to 1.05) \| \| --- \| | | 0.19 | | 0.43 |
| BL Age (years) | 62 | \| 0.97 (0.90 to 1.05) \| \| --- \| | | 0.18 | | 0.44 |
| BL RF (%) | 62 | \| 1.02 (1.00 to 1.04) \| \| --- \| | | 0.16 | | 0.03 |
| BL DAS28 | 62 | \| 1.28 (0.87 to 1.88) \| \| --- \| | | 0.17 | | 0.21 |
| BL CRP (mg/mL) | 62 | \| 1.01 (1.00 to 1.02) \| \| --- \| | | 0.17 | | 0.06 |
| BL 66 SJC | 62 | \| 1.00 (0.96 to 1.05) \| \| --- \| | | 0.19 | | 0.93 |
| BL 68 TJC | 62 | \| 0.98 (0.95 to 1.00) \| \| --- \| | | 0.18 | | 0.10 |
| BL Disease Duration (Years) | 62 | \| 0.99 (0.92 to 1.06) \| \| --- \| | | 0.19 | | 0.70 |
| BL HAQ | 62 | \| 0.80 (0.38 to 1.66) \| \| --- \| | | 0.19 | | 0.55 |
| BL MD Global (0-100) | 62 | \| 1.01 (0.98 to 1.05) \| \| --- \| | | 0.18 | | 0.44 |
| BL PT Global (0-100) | 62 | \| 1.01 (0.98 to 1.03) \| \| --- \| | | 0.19 | | 0.67 |
| BL VAS_pain_ (0-100) | 62 | \| 0.99 (0.96 to 1.02) \| \| --- \| | | 0.19 | | 0.52 |

**Supplementary table B:** BL, baseline; CRP, C-reactive protein; DAS28, disease activity score in 28 joints; csDMARD, conventional synthetic disease modifying antirheumatic drug; HAQ, health assessment questionnaire; IR, inadequate responders; MD, medical doctor; MTX, metothrexate; OR, odds ratio; PT, patient; RF, rheumatoid factor; SJC, swollen joint count; TJC, tender joint count; TT, Targeted therapy; VAS, visual analogue scale.
