# Supplementary material for: Most Trial Eligibility Criteria and Patient Baseline Characteristics Do Not Modify Treatment Effect in Trials Using Targeted Therapies for Rheumatoid Arthritis: A Meta-Epidemiological Study
Source: PLoS One. 2015 Sep 11;10(9):e0136982. doi: 10.1371/journal.pone.0136982 (PMC4567072; doi:10.1371/journal.pone.0136982)
Supplement: S3 Table — (DOCX) [file pone.0136982.s004.docx]

**S3 - Supplementary table 3**

**DAS28 remission analysis, omitting all ‘DMARD-naïve’ trials (DAS28-remission-Modified)**

| **Trial eligibility criteria** | | | | |
| --- | --- | --- | --- | --- |
| **VARIABLE:** | **Trials** | **OR (95% CI)** | τ^2^ | **p-interaction** |
| Overall | 30 | 6.87 (5.05 to 9.35) | 0.30 | N.A. |
| **DMARD History** |  |  | 0.29 | 0.31 |
| DMARD Naive | 0 | N.A. |  |  |
| DMARD-IR | 25 | \| 6.47 (4.66 to 8.97) \| \| --- \| |  |  |
| TT-IR | 5 | \| 10.48 (4.37 to 25.12) \| \| --- \| |  |  |
| **csDMARD Handling at Randomisation** |  |  | 0.34 | 0.78 |
| Naive | 0 | N.A. |  |  |
| Not Using | 6 | \| 9.01 (3.52 to 23.05) \| \| --- \| |  |  |
| Continued | 8 | \| 6.89 (3.97 to 11.96) \| \| --- \| |  |  |
| Discontinued | 15 | \| 6.26 (4.01 to 9.76) \| \| --- \| |  |  |
| Not Reported | 1 | \| 13.42 (2.49 to 72.39) \| \| --- \| |  |  |
| **MTX Handling at Randomisation** |  |  | 0.26 | 0.13 |
| Naive | 0 |  |  |  |
| Not Using | 1 | \| 22.33 (2.34 to 213.50) \| \| --- \| |  |  |
| Continued | 26 | \| 7.27 (5.30 to 9.96) \| \| --- \| |  |  |
| Discontinued | 3 | \| 2.92 (1.10 to 7.74) \| \| --- \| |  |  |
| Not Reported | 0 | N.A. |  |  |
| **TT Handling at Randomisation** |  |  | 0.28 | 0.42 |
| Naive | 6 | \| 4.44 (2.29 to 8.61) \| \| --- \| |  |  |
| Not Using | 11 | \| 6.73 (4.01 to 11.31) \| \| --- \| |  |  |
| Continued | 0 | N.A. |  |  |
| Discontinued | 11 | \| 9.06 (5.46 to 15.03) \| \| --- \| |  |  |
| Not Reported | 2 | \| 6.57 (2.21 to 19.54) \| \| --- \| |  |  |
| **Max Disease Duration at Inclusion** |  |  | 0.28 | 0.14 |
| Early Arthritis (≤2years) | 0 | N.A. |  |  |
| Not Reported | 27 | \| 6.51 (4.77 to 8.88) \| \| --- \| |  |  |
| Established Arthritis (>2 years) | 3 | \| 19.37 (4.69 to 79.99) \| \| --- \| |  |  |
| **Min CRP at Inclusion** |  |  | 0.16 | 0.02 |
| 4.5- 7mg/L or more | 8 | \| 3.36 (1.95 to 5.82) \| \| --- \| |  |  |
| 10 mg/L or more | 12 | \| 8.63 (5.85 to 12.72) \| \| --- \| |  |  |
| 15 mg/L or more | 6 | \| 11.59 (5.53 to 24.29) \| \| --- \| |  |  |
| 20 mg/L or more | 2 | \| 11.16 (2.91 to 42.83) \| \| --- \| |  |  |
| no criteria reported | 2 | \| 3.91 (1.73 to 8.82) \| \| --- \| |  |  |
| **Serology** |  |  |  |  |
| Mixed | 30 | N.A. |  |  |
| Only Seropositive | 0 | N.A. |  |  |
| Only Seronegative | 0 | N.A. |  |  |
| **Min required 66 SJC at Inclusion** |  |  | 0.38 | 0.98 |
| ≥3 | 0 | N.A. |  |  |
| ≥4 | 4 | \| 8.59 (3.40 to 21.71) \| \| --- \| |  |  |
| ≥6 | 16 | \| 6.47 (4.20 to 9.99) \| \| --- \| |  |  |
| ≥8 | 2 | \| 6.39 (2.04 to 20.05) \| \| --- \| |  |  |
| ≥9 | 3 | \| 8.07 (2.07 to 31.36) \| \| --- \| |  |  |
| ≥10 | 5 | \| 7.64 (3.37 to 17.29) \| \| --- \| |  |  |
| **Min required 66 TJC at Inclusion** |  |  | 0.37 | 0.79 |
| ≥4 | 4 | \| 8.58 (3.41 to 21.55) \| \| --- \| |  |  |
| ≥6 | 11 | \| 5.19 (2.98 to 9.04) \| \| --- \| |  |  |
| ≥8 | 7 | \| 8.21 (4.58 to 14.72) \| \| --- \| |  |  |
| ≥9 | 3 | \| 8.05 (2.08 to 31.12) \| \| --- \| |  |  |
| ≥10 | 1 | \| 6.51 (1.22 to 34.74) \| \| --- \| |  |  |
| ≥12 | 4 | \| 8.00 (3.17 to 20.23) \| \| --- \| |  |  |
| **Patient baseline characteristics** | | | | |
| **VARIABLE:** | **Trials** | **Coefficient (95% CI)** | τ^2^ | **p-value** |
| BL Female (%) | 30 | \| 0.98 (0.89 to 1.08) \| \| --- \| | 0.31 | 0.73 |
| BL Age (years) | 30 | \| 1.07 (0.87 to 1.31) \| \| --- \| | 0.31 | 0.53 |
| BL RF (%) | 30 | \| 1.03 (0.97 to 1.09) \| \| --- \| | 0.30 | 0.40 |
| BL DAS28 | 30 | \| 1.13 (0.40 to 3.19) \| \| --- \| | 0.31 | 0.81 |
| BL CRP (mg/mL) | 30 | \| 1.02 (0.99 to 1.06) \| \| --- \| | 0.26 | 0.19 |
| BL 66 SJC | 30 | \| 1.04 (0.94 to 1.14) \| \| --- \| | 0.30 | 0.49 |
| BL 68 TJC | 30 | \| 1.01 (0.95 to 1.08) \| \| --- \| | 0.31 | 0.73 |
| BL Disease Duration (Years) | 30 | \| 1.03 (0.87 to 1.23) \| \| --- \| | 0.31 | 0.72 |
| BL HAQ | 30 | \| 0.76 (0.15 to 3.84) \| \| --- \| | 0.31 | 0.74 |
| BL MD Global (0-100) | 30 | \| 1.01 (0.92 to 1.10) \| \| --- \| | 0.32 | 0.89 |
| BL PT Global (0-100) | 30 | \| 1.01 (0.94 to 1.08) \| \| --- \| | 0.31 | 0.77 |
| BL VAS_pain_ (0-100) | 30 | \| 0.98 (0.91 to 1.07) \| \| --- \| | 0.30 | 0.72 |

**Supplementary table C:** BL, baseline; CRP, C-reactive protein; DAS28, disease activity score in 28 joints; csDMARD, conventional synthetic disease modifying antirheumatic drug; HAQ, health assessment questionnaire; IR, inadequate responders; MD, medical doctor; MTX, metothrexate; OR, odds ratio; PT, patient; RF, rheumatoid factor; SJC, swollen joint count; TJC, tender joint count; TT, Targeted therapy; VAS, visual analogue scale.
