# Supplementary material for: Most Trial Eligibility Criteria and Patient Baseline Characteristics Do Not Modify Treatment Effect in Trials Using Targeted Therapies for Rheumatoid Arthritis: A Meta-Epidemiological Study
Source: PLoS One. 2015 Sep 11;10(9):e0136982. doi: 10.1371/journal.pone.0136982 (PMC4567072; doi:10.1371/journal.pone.0136982)
Supplement: S4 Table — (PDF) [file pone.0136982.s005.pdf]

S4 Table: Dataset

Data on all eligible trials. ID number corresponds to reference number.(1-50)(51-62).

ID number	Name of Biological Agent	Name of Co-allocation	DMARD History	csDMARD Handling at Randomisation	MTX Handling at Randomisation	TT Handling at Randomisation	Max Disease Duration	Min 66 SJC	Min 68 TJC	Min CRP	Min ESR	RF status of all trial patients	Baseline female percentage	Baseline age	Baseline RF percentage	Baseline anti-CCP percentage	Baseline DAS28	Baseline CRP	Baseline ESR	Baseline 66 SJC	Baseline 68 TJC	Baseline RA duration	Baseline MD global (VAS)	Baseline PT global (VAS)	Baseline pain (VAS)	Baseline HAQ	n, randomised in active arm	n, randomised in control arm	Outcome week	Active arm: n, achieving ACR20	Active arm: total in ACR20 analysis	Active arm: n, achieving DAS28-remission	Active arm: total in DAS28-remission analysis	Control arm: n, achieving ACR20	Control arm: total in ACR20 analysis	Control arm: n, achieving DAS28-remission	Control : total in DAS28-remission analysis	Risk of Bias§
1	ABA	PLA	TT IR	CON	CON	DIS	NR	10	12	10 mg/mL<	NR	Mix	78.0	53.2	73.2	NR	6.5	44.0	NR	22.4	31.7	11.9	68.3	69.4	70.5	1.8	262	131	24	129	256	26	256	26	133	1	133	L/L/L/L/L
2	ABA	PLA	DMARD IR	DIS	DIS	DIS	ES	10	12	NR	28	Mix	75.0	49.9	80.7*	NR	7.0#	32.0	32.8	23.7	30.8	3.3	73.2	72.2	70.2	1.5*	32	32	12	17	32	NR	NR	10	32	NR	NR	L/L/L/L/L
3	ABA	MTX	DMARD IR	DIS	CON	NR	NR	10	12	10 mg/mL<	NR	Mix	79.1	51.1	80.7	NR	6.4	31.3	NR	21.6	31.4	8.6	67.8	62.7	64.2	1.7	433	219	26	288	424	63	424	85	214	6	214	L/L/L/L/L

Trial eligibility criteria and patient baseline characteristics

4	TOC	MTX	DMARD IR	DIS	CON	DIS	NR	10	10	10 mg/mL<	28	Mix	77.0	50.5	87.9	NR	6.5	28.0	41.0	16.7	23.8	10.9	63.6	65.4	60.4	1.5*	50	49	16	36	49	17	49	20	49	4	49	L/L/L/L/L/L
5	TOC	MTX	DMARD IR	DIS	CON	DIS	NR	6	8	10 mg/mL<	28	Mix	82.5	52.4	82.5	NR	6.6	22.5	46.4	17.0	28.6	9.2	62.9	62.9	55.5	1.5	399	392	24	224	398	105	315	106	393	8	212	L/L/L/L/U/L
6	TOC	PLA	DMARD IR	CON	CON	NU	NR	6	8	10 mg/mL<	28	Mix	82.0	53.3	80.7*	NR	6.7	26.0	48.5	19.4	29.8	6.9	63.7	66.0	58.3	1.5	805	415	24	488	803	241	803	101	413	24	413	L/L/L/L/L/L
7	TOC	MTX	DMARD IR	DIS	CON	DIS	NR	6	8	10 mg/mL<	28	Mix	81.5	50.7	77.0	NR	6.8	25.0	50.5	20.1	32.3	7.6	63.9	64.2	58.6	1.6	205	204	24	120	205	47	171	54	204	2	121	L/L/L/L/L/L
8	ABA	MTX	DMARD IR	DIS	CON	NAIVE	NR	10	12	10 mg/mL<	NR	Mix	85.0	49.2	86.3	NR	6.9	29.3	48.4	20.8	31.1	8.1	63.6*	63.3*	60.2*	1.8	156	110	28	104	156	18	156	46	110	3	110	L/L/L/L/L/L
9	ABA	MTX	DMARD IR	DIS	CON	DIS	NR	10	12	10 mg/mL<	NR	Mix	70.4	55.2	94.4	NR	6.5¤	30.5	NR	21.6	30.0	9.3	62.7	59.8	63.3	1.0	115	119	26	69	115	NR	NR	42	119	NR	NR	L/L/L/L/L/L
10	TOC	MTX	TT IR	DIS	CON	DIS	NR	6	8	10 mg/mL<	28	Mix	81.6	53.7	77.1	NR	6.8	32.3	51.7	18.9	31.1	12.0	66.9	70.5	64.4	1.7	175	160	24	85	170	40	132	16	158	1	64	L/L/L/L/L/L

11	INF	MTX	DMARD IR	NU	CON	NAIVE	NR	6	6	20 mg/mL<	28	Mix	80.5	53.5	80.5	NR	6.4¤	30.5	NR	19.0	28.0	8.7	63.0	64.0	68.5	1.8	86	88	30	42	83	NR	NR	17	84	NR	NR	L/L/L/L/L
12	INF	MTX	DMARD naive	NAIVE	NAIVE	NAIVE	EA	6*	8*	20 mg/mL<	NR	Mix	81*	52.2	65.0	NR	6.6¤	42.0	NR	19.2*	28.3*	8.7*	63.6*	63.3*	60.2*	1.3	10	10	14	6	10	NR	NR	2	10	NR	NR	U/L/L/H/L
13	RIT	MTX	TT IR	DIS	CON	DIS	NR	8	8	15 mg/mL<	28	Mix	81.0	52.4	79.0	NR	6.9	37.4	48.2	23.2	33.5	11.9	63.6*	63.3*	60.2*	1.9	311	209	24	152	298	45	298	36	201	4	201	L/L/L/L/L
14	RIT	MTX	DMARD IR	DIS	CON	NAIVE	NR	8	8	4.5-7 mg/mL<	28	Mix	83.4	51.7	74.3	NR	6.5	24*	NR	20.2	29.5	7.0	63.6*	63.3*	60.2*	1.5*	172	172	24	86	170	16	170	40	172	4	172	L/L/L/L/L
15	RIT	MTX	DMARD IR	NU	CON	NR	NR	8	8	15 mg/mL<	28	Pos	77.5	54.0	100.0	NR	6.9	30.5	52.5	21.0	32.0	11.5	63.6*	63.3*	60.2*	1.5*	40	40	24	29	40	NR	NR	15	40	NR	NR	L/L/L/L/L
16	RIT	MTX	DMARD IR	DIS	CON	DIS	NR	8	8	15 mg/mL<	28	Pos	80.0	51.1	100.0	NR	6.8	31.5	40.5	21.5	33.5	10.1	63.6*	63.3*	60.2*	1.5*	128	128	24	66	122	NR	NR	34	122	NR	NR	L/L/L/L/L
17	ETA	MTX	DMARD naive	NU	NU	NAIVE	ES	10	12	20 mg/mL<	28	Mix	76.5	52.8	73.5	NR	5.5	27.8	NR	22.4	33.7	6.8	63.6*	63.3*	60.2*	1.5*	229	229	24	187	231	62	205	166	228	26	193	L/L/L/L/L

18	ETA	MTX	DMARD IR	DIS	CON	NR	NR	6	6	NR	NR	Mix	84.3	49.7	86.0	NR	6.6#	23.3	28.7	19.0	28.0	13.0	61.7	60.0	52.0	1.5	59	30	24	42	59	NR	NR	8	30	NR	NR	L/L/L/L/L
19	ADA	PLA	DMARD IR	CON	CON	NR	NR	6	9	NR	NR	Mix	79.4	55.4	62.9	NR	6.1¤	15.0	NR	21.1	27.5	10.4	59.8	53.4	55.4	1.4	318	318	24	168	318	NR	NR	111	318	NR	NR	L/L/L/L/L
20	ADA	MTX	DMARD IR	DIS	CON	NAIVE	NR	6	9	10 mg/mL<	NR	Mix	74.7	51.2	85.5	NR	6.1¤	18.0	NR	19.2	27.7	11.0	61.7	53.5	56.1	1.5	207	200	24	131	207	NR	NR	59	200	NR	NR	L/L/L/L/L
21	ADA	MTX	DMARD IR	DIS	CON	NAIVE	NR	6	9	NR	NR	Mix	78.3	56.6	80.7*	NR	6.3¤	25.8	NR	17.1	28.3	11.7	58.8	57.4	55.0	1.6	67	62	24	45	67	NR	NR	9	62	NR	NR	L/L/L/L/L
22	ADA	PLA	DMARD IR	DIS	DIS	NU	NR	10	12	20 mg/mL<	28	Mix	78.5	53.1	80.7	NR	7.1	54.8	55.9	20.2	34.6	11.1	67.7	72.2	70.1	1.9	113	110	26	52	113	NR	NR	21	110	NR	NR	L/L/L/L/L
23	ETA	PLA	DMARD IR	DIS	DIS	NR	NR	10	12	20 mg/mL<	28	Mix	75.0	52.0	79.0	NR	7.2#	44.0	37.0	25.0	34.0	11.5	69.0	69.5	66.0	1.7	81	83	13	48	78	NR	NR	18	80	NR	NR	L/L/L/L/L
24	GOL	MTX	DMARD naive	NU	NAIVE	NAIVE	NR	4	4	15 mg/mL<	28	Mix	84.3	49.7	80.7*	NR	6.2	25.0	NR	15.4	28.2	3.2	61.0	6.0	6.3	1.5	159	160	24	98	159	40	159	79	160	18	160	L/L/L/L/L

Trial eligibility criteria and patient baseline characteristics

25	GOL	MTX	DMARD IR	NU	CON	NAIVE	NR	4	4	15 mg/mL<	28	Mix	81.6	52.0	83.3	80.7	6.1	8.8	NR	12.4	23.0	5.7	58.6	55.8	58.6	1.3	89	133	14	49	89	14	89	44	133	2	133	L/L/L/L/L/L
26	GOL	PLA	TT IR	CON	CON	NU	NR	4	4	NR	NR	Mix	79.5	54.5	72.5	72.0	6.3	9.0	NR	14.0	26.5	9.7	63.0	66.5	69.5	1.7	153	155	14	54	153	13	153	28	155	1	155	L/L/L/L/L/L
27	ADA	PLA	DMARD IR	DIS	DIS	NAIVE	NR	10	12	20 mg/mL<	NR	Mix	78.1	55.2	87.6	NR	6.6¤	61.8	NR	19.2	24.1	9.2	75.2	68.0	65.5	1.5	91	87	24	40	91	NR	NR	12	87	NR	NR	L/L/L/L/L/L
28	CER	PLA	DMARD IR	DIS	DIS	NAIVE	NR	9	9	10 mg/mL<	28	Mix	83.7	48.7	100.0	NR	6.3	11.5	33.2	20.6	29.0	9.5	72.0	66.0	56.5	1.5	111	109	24	50	111	NR	NR	10	109	NR	NR	L/L/L/L/L/L
29	ETA	SSZ	DMARD IR	NU	NU	NAIVE	ES	6	10	20 mg/mL<	28	Mix	80.8	51.5	80.7*	NR	6.9#	11.6	38.4	19.2	31.3	6.2	63.6*	63.3*	58.7	1.6	104	52	24	74	100	NR	NR	14	50	NR	NR	L/L/L/L/L/L
30	ADA	PLA	DMARD IR	DIS	DIS	NU	NR	10	12	20 mg/mL<	28	Mix	81.0	51.4	87.5	NR	7.1	59.5	52.7	19.5	31.0	9.7	65.1	74.6	72.9	1.7	70	70	12	40	70	NR	NR	7	70	NR	NR	L/L/L/L/L/L
31	TOC	PLA	DMARD IR	DIS	DIS	NR	NR	6	6	10 mg/mL<	30	Mix	78.6	54.5	80.7	NR	6.6#	50.0	68.0	13.8	18.0	8.3	63.6*	63.3*	60.2*	1.5*	55	54	12	43	55	NR	NR	6	53	NR	NR	L/L/L/L/L/L

Trial eligibility criteria and patient baseline characteristics

32	TOC	PLA	DMARD IR	CON	CON	DIS	NR	6	6	10 mg/mL<	28	Mix	81.0	55.4	80.7*	NR	6.5	18.2	46.4	19.8	29.9	8.6	62.4	62.1	56.3	1.5*	412	207	24	183	409	157	409	52	205	3	205	L/L/L/L/L/L
33	RIT	MTX	DMARD naive	NU	NAIVE	NR	ES	8	8	10 mg/mL<	NR	Mix	81.0	48.0	86.0	NR	7.1	31.0	NR	20.8	32.9	0.4	63.6*	63.3*	60.2*	1.8	251	252	24	183	250	NR	NR	147	250	NR	NR	L/L/L/L/L/L
34	GOL	MTX	DMARD IR	NU	CON	NAIVE	NR	6	6	15 mg/mL<	28	Mix	80.0	54.5	80.7*	NR	6.4	20.5	NR	13.5	25.0	6.9	60.5	67.5	70.0	1.5	35	35	16	21	35	2	35	13	35	0	35	L/L/L/L/U/L
35	ETA	MTX	DMARD IR	DIS	CON	DIS	NR	10	10	NR	NR	Mix	86.5	49.2	80.7*	NR	6.1¤	15.2	NR	23.0	21.2	8.7*	77.5	68.0	56.4	1.1	29	29	12	26	29	NR	NR	10	29	NR	NR	U/L/U/U/L/L
36	INF	MTX	DMARD IR	NU	CON	NR	NR	6	6	20 mg/mL<	28	Mix	78.1	55.2	80.7*	NR	6.0¤	41.5	NR	14.3	18.4	8.3	63.6*	63.3*	60.2*	1.5*	50	50	14	30	49	NR	NR	11	47	NR	NR	L/L/L/L/L/L
37	CER	MTX	DMARD IR	DIS	CON	NU	ES	9	9	15 mg/mL<	30	Mix	82.9	51.7	80.7	NR	6.9	16.0	44.0	21.5	30.5	6.1	63.6*	63.3*	60.2*	1.5*	393	199	24	228	388	NR	NR	27	198	NR	NR	L/L/L/L/L/L
38	CER	MTX	DMARD IR	NU	CON	NU	ES	9	9	15 mg/mL<	30	Mix	83.9	52.0	77.7	NR	6.8	14.0	42.7	21.0	30.2	5.9	64.8	61.5	61.2	1.6	246	127	24	141	246	23	246	11	127	1	127	L/L/L/L/H/L

Trial eligibility criteria and patient baseline characteristics

45	44	43	42	41	40	39
CER	TOF	TOF	GOL	ABA	ADA	INF
MTX	PLA	PLA	PLA	MTX	MTX	MTX
DMARD IR	DMARD IR	DMARD IR	DMARD IR	DMARD naive	DMARD IR	DMARD IR
DIS	DIS	DIS	DIS	NU	DIS	CON
CON	DIS	DIS	DIS	NAIVE	CON	CON
NAIVE	NU	DIS	NR	NR	NAIVE	NAIVE
NR	NR	NR	NR	EA	NR	NR
9	6	6	6	10	6	6
9	6	6	6	12	9	6
10 mg/mL<	4.5-7 mg/mL<	4.5-7 mg/mL<	20 mg/mL<	4.5-7 mg/mL<	NR	NR
28	28	28	28	NR	NR	NR
Mix	Mix	Mix	Mix	Pos	Mix	Mix
69.2	88.0	85.5	81.1	77.6	78.7	81.6
54.3	53.5	51.4	52.6	49.9	53.0	52.5
76.1	75.9	80.7*	80.7*	96.4	87.2	81.7
NR	NR	NR	NR	89.0	NR	NR
6.2	6.6	6.7	5.9	6.3	6.4	5.7¤
12.5	24.0	21.2	23.6	33.5	21.0	14.0
25.1	46.8	52.4	NR	NR	NR	NR
22.5	17.1	16.6	12.9	22.4	22.5	15.0
30.0	26.5	29.2	15.2	31.1	33.7	22.0
9.6	9.6	7.9	8.7	0.5	6.7	8.1
71.0	64.3	63.6*	58.6	63.6*	81.4	62.5
66.0	64.2	63.3*	54.3	63.3*	72.0	56.5
58.7	62.1	60.2*	55.4	60.2*	64.8	60.0
1.4	1.5	1.5	1.0	1.7	1.7	1.5
126	50	244	102	256	35	360
121	59	122	110	255	12	363
24	12	13	14	12	12	22
56	29	144	51	164	19	199
124	49	241	101	256	35	343
9	6	14	13	41	NR	106
97	49	229	97	256	NR	343
27	13	32	20	134	4	87
119	59	120	105	253	12	341
2	2	5	2	20	NR	48
65	59	104	94	253	NR	341
L/L/L/L/L/L	L/L/L/L/L/L	L/L/L/L/L/L	L/L/L/L/L/L	L/L/L/L/L/L	U/L/L/L/L/L	L/L/L/L/H/L

46	CER	PLA	DMARD IR	CON	NU	NU	ES	6	6	20 mg/mL<	28	Mix	74.4	55.7	87.4	NR	6.2	16.5	50.0	14.6	16.9	5.6	60.9	56.8	56.8	1.1	116	114	12	78	116	19	116	17	114	1	114	L/L/L/L/L/L
47	CER	MTX	DMARD IR	NU	CON	NU	ES	9	9	15 mg/mL<	30	Mix	84.9	51.2	86.2	NR	6.3	15.0	46.9	17.0	19.3	5.7	63.3	55.3	58.2	1.1	82	77	12	63	82	13	82	22	77	0	77	L/L/L/L/L/L
48	ADA	MTX	DMARD naive	NU	NAIVE	NAIVE	EA	6	8	15 mg/mL<	28	Mix	74.0	50.5	88.0	84.0	6.0	28.5	NR	12.5	16.0	4.3	62.5	63.5	65.0	1.6	515	517	26	361	515	175	515	295	517	88	517	L/L/L/L/L/L
49	ABA	MTX	DMARD IR	DIS	CON	DIS	NR	10	12	10 mg/mL<	NR	Mix	79.5	53.4	80.7*	NR	6.0	33.9	NR	17.1	21.7	7.3	63.6*	65.4	60.2*	1.4	62	66	24	47	61	15	61	14	66	1	66	L/L/L/L/L/L
50	GOL	MTX	DMARD IR	NR	CON	NU	NR	4	4	15 mg/mL<	28	Mix	83.9	50.8	80.7*	NR	5.6	20.5	NR	11.6	13.2	8.7	56.2	48.4	50.9	1.0	89	90	14	62	86	27	86	24	88	3	88	L/L/L/L/L/L
51	TOF	MTX	DMARD IR	NU	CON	NU	NR	6	6	4.5-7 mg/mL<	28	Mix	82.0	53.3	66.6	70.7	6.6	15.3	48.4	16.7	28.1	7.7	63.6*	63.3*	60.2*	1.5	204	108	26	101	196	11	177	30	106	1	92	L/L/L/L/L/L
52	GOL	MTX	DMARD IR	NU	CON	NAIVE	NR	6	6	10 mg/mL<	NR	Pos	81.6	52.7	80.7*	NR	6.0	19.0	NR	12.0	23.3	4.7	63.6*	63.3*	60.2*	1.6	395	197	14	231	395	NR	NR	49	197	NR	NR	L/L/L/L/L/L

53	CER	PLA	DMARD IR	CON	CON	NU	NR	6	8	10 mg/mL<	28	Mix	78.0	55.1	74.4	66.3	6.4	9.2	37.6	17.0	23.1	5.6	63.6*	63.3*	60.2*	1.5	851	212	12	435	851	136	851	55	212	12	212	L/L/L/L/L/L
54	TOF	MTX	DMARD IR	DIS	CON	DIS	NR	6	6	4.5-7 mg/mL<	28	Mix	85.4	50.3	85.5	NR	6.0	19.5	NR	14.7	17.1	8.4	59.4	56.1	53.3	1.3	28	28	12	26	27	NR	NR	4	28	NR	NR	L/L/L/L/L/L
55	ADA	MTX	DMARD IR	DIS	CON	NR	NR	6	9	NR	NR	Mix	90.6	49.1	79.7	NR	5.0¤	2.4	NR	12.5	19.7	6.8	63.8	61.4	58.5	1.4	65	63	24	40	65	NR	NR	23	63	NR	NR	L/L/L/L/L/L
56	INF	MTX	DMARD naive	NU	NAIVE	NR	EA	6	8	NR	NR	Mix	68.9	51.8	65.6	58.0	5.3	36.9	NR	11.4	13.8	0.4	63.6*	63.3*	60.2*	1.4	15	14	22	13	15	NR	NR	4	14	NR	NR	L/L/H/L/H/L
57	TOF	MTX	DMARD IR	NU	CON	NU	NR	6	6	4.5-7 mg/mL<	28	Mix	84.4	53.4	76.0	85.0	6.3	14.9	50.4	14.1	23.7	9.0	58.2	56.8	57.3	1.4	321	160	26	159	309	19	265	39	154	2	129	L/L/L/L/H/L
58	TOF	MTX	TT IR	DIS	CON	DIS	NR	6	6	4.5-7 mg/mL<	28	Mix	82.7	54.9	63.1	72.1	6.5	18.0	47.3	16.7	28.3	12.2	64.9	63.3	63.2	1.6	133	132	13	55	132	8	119	32	131	2	120	L/L/L/L/L/L
59	TOF	MTX	DMARD IR	DIS	CON	DIS	NR	6	6	4.5-7 mg/mL<	28	Mix	80.7	52.5	82.9	NR	6.1	18.4	NR	14.9	21.5	9.1	53.3	57.4	53.1	1.3	71	69	12	40	71	10	60	25	69	4	60	L/L/L/L/L/L

Trial eligibility criteria and patient baseline characteristics

60	ADA	MTX	DMARD naive	DIS	NAIVE	NAIVE	EA	8	10	15 mg/mL<	28	Mix	81.4	54.0	84.4	84.1	6.6	30.0	60.8	16.9	13.2	0.3	66.0	65.2	60.2*	1.2	171	163	26	129	171	53	171	92	163	24	163	L/L/L/L/L
61	TOF	PLA	DMARD IR	CON	CON	NU	NR	4	4	4.5-7 mg/mL<	28	Mix	81.7	52.5	73.5	69.2	6.3	17.4	50.4	14.4	24.8	8.7	59.9	58.7	57.1	1.4	318	159	26	164	315	24	284	49	159	4	153	L/L/L/L/L
62	INF	MTX	DMARD IR	NU	CON	NR	NR	3	8	NR	28	Mix	85.0	48.4	80.7*	NR	6.4¤	24*	NR	19.2*	28.3*	7.5	63.6*	63.3*	60.2*	1.5*	87	86	18	66	87	NR	NR	42	86	NR	NR	L/L/L/U/L

ABA, abatacept; ACR20, American College of Rheumatology 20% improvement criteria; ADA, adalimumab; Anti-CCP, anti-cyclic citrullinated peptide; BL, baseline; CER, certolizumab; CON, continued; DAS28, disease activity score in 28 joints; DIS, Discontinued; csDMARD, conventional synthetic disease modifying antirheumatic drugs; ETA, etanercept; GOL, golimumab; HAQ, health assessment questionnaire; INF, infliximab; IR, inadequate responders; MD global, physicians global assessment (0-100); Min, minimum; MTX, metothrexate; NR, Not reported; NU, Not using; OR, odds ratio; PLA, placebo; PT global, patient global assessment; RIT, rituximab; RF, rheumatoid factor; SJC, swollen joint count; TJC, tender joint count; TOC, tocilizumab; TOF, tofacitinib; TT, Targeted therapy; VAS, visual analogue scale (0-100).

*Median values for the dataset imputed

Calculated from DAS28-ESR(4) formula: 0.56*sqrt(TJC28) + 0.28*sqrt(SJC28) + 0.70*Ln(ESR) + 0.014*GH

¤Calculated from DAS28-CRP(4) formula: 0.56*sqrt(TJC28) + 0.28*sqrt(SJC28) + 0.36*ln(CRP+1) + 0.014*GH + 0.96

§Risk of bias rated as L, low; U, unclear; H, high per domain: Allocation concealment/ Blinding of participants and personnel/ Blinding of outcome assessment/ Incomplete outcome data/ Selective reporting

Reference List

(1) Genovese MC, Becker JC, Schiff M, Luggen M, Sherrer Y, Kremer J, et al. Abatacept for rheumatoid arthritis refractory to tumor necrosis factor alpha inhibition. N Engl J Med 2005 Sep 15;353(11):1114-23.

(2) Moreland LW, Alten R, Van den Bosch F, Appelboom T, Leon M, Emery P, et al. Costimulatory blockade in patients with rheumatoid arthritis: a pilot, dose-finding, double-blind, placebo-controlled clinical trial evaluating CTLA-4Ig and LEA29Y eighty-five days after the first infusion. Arthritis Rheum 2002 Jun;46(6):1470-9.

(3) Kremer JM, Genant HK, Moreland LW, Russell AS, Emery P, Abud-Mendoza C, et al. Effects of abatacept in patients with methotrexate-resistant active rheumatoid arthritis: a randomized trial. Ann Intern Med 2006 Jun 20;144(12):865-76.

(4) Maini RN, Taylor PC, Szechinski J, Pavelka K, Broll J, Balint G, et al. Double-blind randomized controlled clinical trial of the interleukin-6 receptor antagonist, tocilizumab, in European patients with rheumatoid arthritis who had an incomplete response to methotrexate. Arthritis Rheum 2006 Sep;54(9):2817-29.

(5) Kremer JM, Blanco R, Brzosko M, Burgos-Vargas R, Halland AM, Vernon E, et al. Tocilizumab inhibits structural joint damage in rheumatoid arthritis patients with inadequate responses to methotrexate: results from the double-blind treatment phase of a randomized placebo-controlled trial of tocilizumab safety and prevention of structural joint damage at one year. Arthritis Rheum 2011 Mar;63(3):609-21.

(6) Genovese MC, McKay JD, Nasonov EL, Mysler EF, da Silva NA, Alecock E, et al. Interleukin-6 receptor inhibition with tocilizumab reduces disease activity in rheumatoid arthritis with inadequate response to disease-modifying antirheumatic drugs: the tocilizumab in combination with traditional disease-modifying antirheumatic drug therapy study. Arthritis Rheum 2008 Oct;58(10):2968-80.

(7) Smolen JS, Beaulieu A, Rubbert-Roth A, Ramos-Remus C, Rovensky J, Alecock E, et al. Effect of interleukin-6 receptor inhibition with tocilizumab in patients with rheumatoid arthritis (OPTION study): a double-blind, placebo-controlled, randomised trial. Lancet 2008 Mar 22;371(9617):987-97.

(8) Schiff M, Keiserman M, Codding C, Songcharoen S, Berman A, Nayiager S, et al. Efficacy and safety of abatacept or infliximab vs placebo in ATTEST: a phase III, multi-centre, randomised, double-blind, placebo-controlled study in patients with rheumatoid arthritis and an inadequate response to methotrexate. Ann Rheum Dis 2008 Aug;67(8):1096-103.

(9) Kremer JM, Westhovens R, Leon M, Di GE, Alten R, Steinfeld S, et al. Treatment of rheumatoid arthritis by selective inhibition of T-cell activation with fusion protein CTLA4Ig. N Engl J Med 2003 Nov 13;349(20):1907-15.

(10) Emery P, Keystone E, Tony HP, Cantagrel A, van VR, Sanchez A, et al. IL-6 receptor inhibition with tocilizumab improves treatment outcomes in patients with rheumatoid arthritis refractory to anti-tumour necrosis factor biologicals: results from a 24-week multicentre randomised placebo-controlled trial. Ann Rheum Dis 2008 Nov;67(11):1516-23.

(11) Maini R, St Clair EW, Breedveld F, Furst D, Kalden J, Weisman M, et al. Infliximab (chimeric anti-tumour necrosis factor alpha monoclonal antibody) versus placebo in rheumatoid arthritis patients receiving concomitant methotrexate: a randomised phase III trial. ATTRACT Study Group. Lancet 1999 Dec 4;354(9194):1932-9.

(12) Quinn MA, Conaghan PG, O'Connor PJ, Karim Z, Greenstein A, Brown A, et al. Very early treatment with infliximab in addition to methotrexate in early, poor-prognosis rheumatoid arthritis reduces magnetic resonance imaging evidence of synovitis and damage, with sustained benefit after infliximab withdrawal: results from a twelve-month randomized, double-blind, placebo-controlled trial. Arthritis Rheum 2005 Jan;52(1):27-35.

(13) Cohen SB, Emery P, Greenwald MW, Dougados M, Furie RA, Genovese MC, et al. Rituximab for rheumatoid arthritis refractory to anti-tumor necrosis factor therapy: Results of a multicenter, randomized, double-blind, placebo-controlled, phase III trial evaluating primary efficacy and safety at twenty-four weeks. Arthritis Rheum 2006 Sep;54(9):2793-806.

(14) Emery P, Deodhar A, Rigby WF, Isaacs JD, Combe B, Racewicz AJ, et al. Efficacy and safety of different doses and retreatment of rituximab: a randomised, placebo-controlled trial in patients who are biological naive with active rheumatoid arthritis and an inadequate response to methotrexate (Study Evaluating Rituximab's Efficacy in MTX iNadequate rEsponders (SERENE)). Ann Rheum Dis 2010 Sep;69(9):1629-35.

(15) Edwards JC, Szczepanski L, Szechinski J, Filipowicz-Sosnowska A, Emery P, Close DR, et al. Efficacy of B-cell-targeted therapy with rituximab in patients with rheumatoid arthritis. N Engl J Med 2004 Jun 17;350(25):2572-81.

(16) Emery P, Fleischmann R, Filipowicz-Sosnowska A, Schechtman J, Szczepanski L, Kavanaugh A, et al. The efficacy and safety of rituximab in patients with active rheumatoid arthritis despite methotrexate treatment: results of a phase IIB randomized, double-blind, placebo-controlled, dose-ranging trial. Arthritis Rheum 2006 May;54(5):1390-400.

(17) Klareskog L, van der Heijde D, de Jager JP, Gough A, Kalden J, Malaise M, et al. Therapeutic effect of the combination of etanercept and methotrexate compared with each treatment alone in patients with rheumatoid arthritis: double-blind randomised controlled trial. Lancet 2004 Feb 28;363(9410):675-81.

(18) Weinblatt ME, Kremer JM, Bankhurst AD, Bulpitt KJ, Fleischmann RM, Fox RI, et al. A trial of etanercept, a recombinant tumor necrosis factor receptor:Fc fusion protein, in patients with rheumatoid arthritis receiving methotrexate. N Engl J Med 1999 Jan 28;340(4):253-9.

(19) Furst DE, Schiff MH, Fleischmann RM, Strand V, Birbara CA, Compagnone D, et al. Adalimumab, a fully human anti tumor necrosis factor-alpha monoclonal antibody, and concomitant standard antirheumatic therapy for the treatment of rheumatoid arthritis: results of STAR (Safety Trial of Adalimumab in Rheumatoid Arthritis). J Rheumatol 2003 Dec;30(12):2563-71.

(20) Keystone EC, Kavanaugh AF, Sharp JT, Tannenbaum H, Hua Y, Teoh LS, et al. Radiographic, clinical, and functional outcomes of treatment with adalimumab (a human anti-tumor necrosis factor monoclonal antibody) in patients with active rheumatoid arthritis receiving concomitant methotrexate therapy: a randomized, placebo-controlled, 52-week trial. Arthritis Rheum 2004 May;50(5):1400-11.

(21) Weinblatt ME, Keystone EC, Furst DE, Moreland LW, Weisman MH, Birbara CA, et al. Adalimumab, a fully human anti-tumor necrosis factor alpha monoclonal antibody, for the treatment of rheumatoid arthritis in patients taking concomitant methotrexate: the ARMADA trial. Arthritis Rheum 2003 Jan;48(1):35-45.

(22) van de Putte LB, Atkins C, Malaise M, Sany J, Russell AS, Van Riel PL, et al. Efficacy and safety of adalimumab as monotherapy in patients with rheumatoid arthritis for whom previous disease modifying antirheumatic drug treatment has failed. Ann Rheum Dis 2004 May;63(5):508-16.

(23) Moreland LW, Schiff MH, Baumgartner SW, Tindall EA, Fleischmann RM, Bulpitt KJ, et al. Etanercept therapy in rheumatoid arthritis. A randomized, controlled trial. Ann Intern Med 1999 Mar 16;130(6):478-86.

(24) Emery P, Fleischmann RM, Moreland LW, Hsia EC, Strusberg I, Durez P, et al. Golimumab, a human anti-tumor necrosis factor alpha monoclonal antibody, injected subcutaneously every four weeks in methotrexate-naive patients with active rheumatoid arthritis: twenty-four-week results of a phase III, multicenter, randomized, double-blind, placebo-controlled study of golimumab before methotrexate as first-line therapy for early-onset rheumatoid arthritis. Arthritis Rheum 2009 Aug;60(8):2272-83.

(25) Keystone EC, Genovese MC, Klareskog L, Hsia EC, Hall ST, Miranda PC, et al. Golimumab, a human antibody to tumour necrosis factor {alpha} given by monthly subcutaneous injections, in active rheumatoid arthritis despite methotrexate therapy: the GO-FORWARD Study. Ann Rheum Dis 2009 Jun;68(6):789-96.

(26) Smolen JS, Kay J, Doyle MK, Landewe R, Matteson EL, Wollenhaupt J, et al. Golimumab in patients with active rheumatoid arthritis after treatment with tumour necrosis factor alpha inhibitors (GO-AFTER study): a multicentre, randomised, double-blind, placebo-controlled, phase III trial. Lancet 2009 Jul 18;374(9685):210-21.

(27) Miyasaka N. Clinical investigation in highly disease-affected rheumatoid arthritis patients in Japan with adalimumab applying standard and general evaluation: the CHANGE study. Mod Rheumatol 2008;18(3):252-62.

(28) Fleischmann R, Vencovsky J, van Vollenhoven RF, Borenstein D, Box J, Coteur G, et al. Efficacy and safety of certolizumab pegol monotherapy every 4 weeks in patients with rheumatoid arthritis failing previous disease-modifying antirheumatic therapy: the FAST4WARD study. Ann Rheum Dis 2009 Jun;68(6):805-11.

(29) Combe B, Codreanu C, Fiocco U, Gaubitz M, Geusens PP, Kvien TK, et al. Etanercept and sulfasalazine, alone and combined, in patients with active rheumatoid arthritis despite receiving sulfasalazine: a double-blind comparison. Ann Rheum Dis 2006 Oct;65(10):1357-62.

(30) van de Putte LB, Rau R, Breedveld FC, Kalden JR, Malaise MG, Van Riel PL, et al. Efficacy and safety of the fully human anti-tumour necrosis factor alpha monoclonal antibody adalimumab (D2E7) in DMARD refractory patients with rheumatoid arthritis: a 12 week, phase II study. Ann Rheum Dis 2003 Dec;62(12):1168-77.

(31) Nishimoto N, Yoshizaki K, Miyasaka N, Yamamoto K, Kawai S, Takeuchi T, et al. Treatment of rheumatoid arthritis with humanized anti-interleukin-6 receptor antibody: a multicenter, double-blind, placebo-controlled trial. Arthritis Rheum 2004 Jun;50(6):1761-9.

(32) Yazici Y, Curtis JR, Ince A, Baraf H, Malamet RL, Teng LL, et al. Efficacy of tocilizumab in patients with moderate to severe active rheumatoid arthritis and a previous inadequate response to disease-modifying antirheumatic drugs: the ROSE study. Ann Rheum Dis 2012 Feb;71(2):198-205.

(33) Tak PP, Rigby WF, Rubbert-Roth A, Peterfy CG, van Vollenhoven RF, Stohl W, et al. Inhibition of joint damage and improved clinical outcomes with rituximab plus methotrexate in early active rheumatoid arthritis: the IMAGE trial. Ann Rheum Dis 2011 Jan;70(1):39-46.

(34) Kay J, Matteson EL, Dasgupta B, Nash P, Durez P, Hall S, et al. Golimumab in patients with active rheumatoid arthritis despite treatment with methotrexate: a randomized, double-blind, placebo-controlled, dose-ranging study. Arthritis Rheum 2008 Apr;58(4):964-75.

(35) Lan JL, Chou SJ, Chen DY, Chen YH, Hsieh TY, Young M, Jr. A comparative study of etanercept plus methotrexate and methotrexate alone in Taiwanese patients with active rheumatoid arthritis: a 12-week, double-blind, randomized, placebo-controlled study. J Formos Med Assoc 2004 Aug;103(8):618-23.

(36) Abe T, Takeuchi T, Miyasaka N, Hashimoto H, Kondo H, Ichikawa Y, et al. A multicenter, double-blind, randomized, placebo controlled trial of infliximab combined with low dose methotrexate in Japanese patients with rheumatoid arthritis. J Rheumatol 2006 Jan;33(1):37-44.

(37) Keystone E, Heijde D, Mason D, Jr., Landewe R, Vollenhoven RV, Combe B, et al. Certolizumab pegol plus methotrexate is significantly more effective than placebo plus methotrexate in active rheumatoid arthritis: findings of a fifty-two-week, phase III, multicenter, randomized, double-blind, placebo-controlled, parallel-group study. Arthritis Rheum 2008 Nov;58(11):3319-29.

(38) Smolen J, Landewe RB, Mease P, Brzezicki J, Mason D, Luijtens K, et al. Efficacy and safety of certolizumab pegol plus methotrexate in active rheumatoid arthritis: the RAPID 2 study. A randomised controlled trial. Ann Rheum Dis 2009 Jun;68(6):797-804.

(39) Westhovens R, Yocum D, Han J, Berman A, Strusberg I, Geusens P, et al. The safety of infliximab, combined with background treatments, among patients with rheumatoid arthritis and various comorbidities: a large, randomized, placebo-controlled trial. Arthritis Rheum 2006 Apr;54(4):1075-86.

(40) Chen DY, Chou SJ, Hsieh TY, Chen YH, Chen HH, Hsieh CW, et al. Randomized, double-blind, placebo-controlled, comparative study of human anti-TNF antibody adalimumab in combination with methotrexate and methotrexate alone in Taiwanese patients with active rheumatoid arthritis. J Formos Med Assoc 2009 Apr;108(4):310-9.

(41) Westhovens R, Robles M, Ximenes AC, Nayiager S, Wollenhaupt J, Durez P, et al. Clinical efficacy and safety of abatacept in methotrexate-naive patients with early rheumatoid arthritis and poor prognostic factors. Ann Rheum Dis 2009 Dec;68(12):1870-7.

(42) Takeuchi T, Harigai M, Tanaka Y, Yamanaka H, Ishiguro N, Yamamoto K, et al. Golimumab monotherapy in Japanese patients with active rheumatoid arthritis despite prior treatment with disease-modifying antirheumatic drugs: results of the phase 2/3, multicentre, randomised, double-blind, placebo-controlled GO-MONO study through 24 weeks. Ann Rheum Dis 2013 Sep 1;72(9):1488-95.

(43) Fleischmann R, Kremer J, Cush J, Schulze-Koops H, Connell CA, Bradley JD, et al. Placebo-controlled trial of tofacitinib monotherapy in rheumatoid arthritis. N Engl J Med 2012 Aug 9;367(6):495-507.

(44) Fleischmann R, Cutolo M, Genovese MC, Lee EB, Kanik KS, Sadis S, et al. Phase IIb dose-ranging study of the oral JAK inhibitor tofacitinib (CP-690,550) or adalimumab monotherapy versus placebo in patients with active rheumatoid arthritis with an inadequate response to disease-modifying antirheumatic drugs. Arthritis Rheum 2012 Mar;64(3):617-29.

(45) Choy E, McKenna F, Vencovsky J, Valente R, Goel N, Vanlunen B, et al. Certolizumab pegol plus MTX administered every 4 weeks is effective in patients with RA who are partial responders to MTX. Rheumatology (Oxford) 2012 Jul;51(7):1226-34.

(46) Yamamoto K, Takeuchi T, Yamanaka H, Ishiguro N, Tanaka Y, Eguchi K, et al. Efficacy and safety of certolizumab pegol without methotrexate co-administration in Japanese patients with active rheumatoid arthritis: the HIKARI randomized, placebo-controlled trial. Mod Rheumatol 2014 Jul;24(4):552-60.

(47) Yamamoto K, Takeuchi T, Yamanaka H, Ishiguro N, Tanaka Y, Eguchi K, et al. Efficacy and safety of certolizumab pegol plus methotrexate in Japanese rheumatoid arthritis patients with an inadequate response to methotrexate: the J-RAPID randomized, placebo-controlled trial. Mod Rheumatol 2014 Sep;24(5):715-24.

(48) Kavanaugh A, Fleischmann RM, Emery P, Kupper H, Redden L, Guerette B, et al. Clinical, functional and radiographic consequences of achieving stable low disease activity and remission with adalimumab plus methotrexate or methotrexate alone in early rheumatoid arthritis: 26-week results from the randomised, controlled OPTIMA study. Ann Rheum Dis 2013 Jan;72(1):64-71.

(49) Takeuchi T, Matsubara T, Nitobe T, Suematsu E, Ohta S, Honjo S, et al. Phase II dose-response study of abatacept in Japanese patients with active rheumatoid arthritis with an inadequate response to methotrexate. Mod Rheumatol 2013 Mar;23(2):226-35.

(50) Tanaka Y, Harigai M, Takeuchi T, Yamanaka H, Ishiguro N, Yamamoto K, et al. Golimumab in combination with methotrexate in Japanese patients with active rheumatoid arthritis: results of the GO-FORTH study. Ann Rheum Dis 2012 Jun;71(6):817-24.

(51) van Vollenhoven RF, Fleischmann R, Cohen S, Lee EB, Garcia Meijide JA, Wagner S, et al. Tofacitinib or adalimumab versus placebo in rheumatoid arthritis. N Engl J Med 2012 Aug 9;367(6):508-19.

(52) Weinblatt ME, Bingham CO, III, Mendelsohn AM, Kim L, Mack M, Lu J, et al. Intravenous golimumab is effective in patients with active rheumatoid arthritis despite methotrexate therapy with responses as early as week 2: results of the phase 3, randomised, multicentre, double-blind, placebo-controlled GO-FURTHER trial. Ann Rheum Dis 2013 Mar;72(3):381-9.

(53) Weinblatt ME, Fleischmann R, Huizinga TW, Emery P, Pope J, Massarotti EM, et al. Efficacy and safety of certolizumab pegol in a broad population of patients with active rheumatoid arthritis: results from the REALISTIC phase IIIb study. Rheumatology (Oxford) 2012 Dec;51(12):2204-14.

(54) Tanaka Y, Suzuki M, Nakamura H, Toyoizumi S, Zwillich SH. Phase II study of tofacitinib (CP-690,550) combined with methotrexate in patients with rheumatoid arthritis and an inadequate response to methotrexate. Arthritis Care Res (Hoboken) 2011 Aug;63(8):1150-8.

(55) Kim H-Y, Lee S-K, Song YW, Yoo D-H, Koh E-M, Yoo B, et al. A randomized, double-blind, placebo-controlled, phase III study of the human anti-tumor necrosis factor antibody adalimumab administered as subcutaneous injections in Korean rheumatoid arthritis patients treated with methotrexate. APLAR Journal of Rheumatology 2007;10(1):April.

(56) Durez P, Malghem J, Nzeusseu TA, Depresseux G, Lauwerys BR, Westhovens R, et al. Treatment of early rheumatoid arthritis: a randomized magnetic resonance imaging study comparing the effects of methotrexate alone, methotrexate in combination with infliximab, and methotrexate in combination with intravenous pulse methylprednisolone. Arthritis Rheum 2007 Dec;56(12):3919-27.

(57) van der Heijde D, Tanaka Y, Fleischmann R, Keystone E, Kremer J, Zerbini C, et al. Tofacitinib (CP-690,550) in patients with rheumatoid arthritis receiving methotrexate: twelve-month data from a twenty-four-month phase III randomized radiographic study. Arthritis Rheum 2013 Mar;65(3):559-70.

(58) Burmester GR, Blanco R, Charles-Schoeman C, Wollenhaupt J, Zerbini C, Benda B, et al. Tofacitinib (CP-690,550) in combination with methotrexate in patients with active rheumatoid arthritis with an inadequate response to tumour necrosis factor inhibitors: a randomised phase 3 trial. Lancet 2013 Feb 9;381(9865):451-60.

(59) Kremer JM, Cohen S, Wilkinson BE, Connell CA, French JL, Gomez-Reino J, et al. A phase IIb dose-ranging study of the oral JAK inhibitor tofacitinib (CP-690,550) versus placebo in combination with background methotrexate in patients with active rheumatoid arthritis and an inadequate response to methotrexate alone. Arthritis Rheum 2012 Apr;64(4):970-81.

(60) Takeuchi T, Yamanaka H, Ishiguro N, Miyasaka N, Mukai M, Matsubara T, et al. Adalimumab, a human anti-TNF monoclonal antibody, outcome study for the prevention of joint damage in Japanese patients with early rheumatoid arthritis: the HOPEFUL 1 study. Ann Rheum Dis 2014 Mar;73(3):536-43.

(61) Kremer J, Li ZG, Hall S, Fleischmann R, Genovese M, Martin-Mola E, et al. Tofacitinib in combination with nonbiologic disease-modifying antirheumatic drugs in patients with active rheumatoid arthritis: a randomized trial. Ann Intern Med 2013 Aug 20;159(4):253-61.

(62) Zhang F-C, Hou Y, Huang F, Wu D-H, Bao C-D, Ni L-Q, et al. Infliximab versus placebo in rheumatoid arthritis patients receiving concomitant methotrexate: A preliminary study from China. APLAR Journal of Rheumatology 2006;9(2):August.
